# A systematic review of reach, adoption, implementation and maintenance of Internet-based interventions to prevent eating disorders in adults

**DOI:** 10.1093/eurpub/ckab044

**Published:** 2021-07-07

**Authors:** Barbara Nacke, Michael Zeiler, Stefanie Kuso, Lisa M Klesges, Corinna Jacobi, Karin Waldherr

**Affiliations:** 1 Technische Universität Dresden, Institute of Clinical Psychology and Psychotherapy, Dresden, Germany; 2 Department for Child and Adolescent Psychiatry, Eating Disorder Unit, Medical University of Vienna, Vienna, Austria; 3 Ferdinand Porsche FernFH—Distance Learning University of Applied Sciences, Wiener Neustadt, Austria; 4 School of Public Health, University of Memphis, Memphis, TN, USA

## Abstract

**Background:**

There is a growing body of research and evidence for the efficacy of Internet-based eating disorder (ED) prevention interventions for adults. However, much less is known about the reach, adoption, implementation and maintenance of these interventions. The RE-AIM (reach, efficacy/effectiveness, adoption, implementation, maintenance) model provides a framework to systematically assess this information.

**Methods:**

A literature search was conducted in PubMed, Web of Science and PsycINFO for articles published between 2000 and 2019. Additionally, reference lists of the studies included and existing reviews published until the end of 2020 were searched. Sixty original articles describing 54 individual studies fulfilled inclusion criteria. Data were extracted for a total of 43 RE-AIM indicators for each study. Fostering and hindering factors for reach, adoption, implementation and maintenance were assessed qualitatively.

**Results:**

Overall reporting rates were best for the RE-AIM dimensions reach (62.6%), implementation (57.0%) and effectiveness (54.2%), while adoption (24.2%) and maintenance (21.5%) had comparatively low overall reporting rates. Reporting on indicators of internal validity, such as sample size, effects or description of interventions was better than indicators relevant for dissemination and implementation in real-world settings, e.g. characteristics of non-participants, characteristics and representativeness of settings, and data to estimate cost.

**Conclusions:**

Because most Internet-based ED prevention interventions are provided in a research-funded context, little is known about their public health impact. Better reporting of factors determining external validity is needed to inform dissemination and implementation of these interventions.

## Introduction

Disordered eating, body dissatisfaction and poor exercise habits can impair not only young women but also adults of all age groups.^1^^,^^2^ Previous research has established a number of risk factors and correlates concerning disordered eating, full syndrome eating disorders (EDs) and overweight. These include dieting,^3^^,^^4^ loss of control eating, body dissatisfaction/elevated weight and shape concerns, negative self-evaluation,^4^^,^^5^ compensatory behaviours in general as well as purging.^4^ Variable risk factors for EDs can be affected by preventive interventions. There is considerable evidence for the efficacy of Internet-based ED prevention programmes, as they have been shown to decrease ED-related symptoms and risk factors, such as shape and weight concerns, body dissatisfaction, dieting, bingeing and purging behaviours as well as internalization of the thin ideal.^6–10^ As these programmes are rarely translated into routine practice outside of research funding,^11^ few studies focused on outcomes other than efficacy allowing to evaluate their dissemination potential and public health impact, e.g. reach of the programmes, uptake by settings, implementation fidelity and sustainability.^12^ Likewise, previous literature reviews primarily focussed on the efficacy of these interventions. However, optimizing factors such as reach, engagement, implementation or maintenance could ultimately improve intervention outcomes and consequently public health impact, as the benefits of ED prevention interventions on a population level will increase as more people utilize them.^12–14^ Therefore, it is important to report these factors both in controlled research studies and in more practice-oriented studies to improve their chances for successful implementation in real-world settings. As Internet-based prevention has high potential for scaling,^11^^,^^15^ this systematic review aims to expand the existing base of evidence of Internet-based prevention programmes for adults beyond effectiveness measures and to identify research gaps.

For this purpose, we will apply the RE-AIM model,^12^ a framework that guides the evaluation of measures of both internal and external validity and proposes reporting standards for real-world effectiveness of interventions. This framework will be used to systematically review the reach, adoption, implementation and maintenance of Internet-based ED prevention programmes for adults. Another review on ED prevention programmes for adolescents using the RE-AIM framework has been recently published, identifying publication gaps for factors of external validity relevant to dissemination.^16^ Considering the extensive research, including recent systematic reviews and meta-analyses,^6–10^ on the efficacy of ED prevention programmes for adults, we will not include the size of intervention effects in this review.

## Methods

### Study design

The RE-AIM framework^12^ will be used to systematically review the degree to which indicators for the reach of participants (e.g. intended target group and representativeness of participants), efficacy/effectiveness (effects of the intervention on specified outcomes), adoption (e.g. number and characteristics of settings that offer an intervention to the target population), implementation (e.g. fidelity of intervention delivery) and maintenance (sustained engagement on an individual and organizational level) of Internet-based ED prevention programmes for adults are reported in the literature. A systematic review was conducted and reporting follows the PRISMA guidelines.^17^ The review was not registered in advance. As the literature search for the present review was conducted jointly with a recently published review on Internet-based interventions for adolescents, the methods for literature search, extraction and exclusion described below parallel the methods published in the aforementioned review.^16^

### Information sources and search strategy

The literature search was conducted using three electronic databases (PubMed, PsycINFO and Web of Science) and included publications dating from 1 January 2000 to 9 April 2019. We did not search for literature published before 2000 as previous reviews suggest that hardly any relevant studies have been published before. Keywords referred to (prevention) programmes, digital or Internet-based technology and to the topic of EDs, eating behaviours and body image (see Supplementary table S1 for the search syntax). In addition, 32 narrative and systematic reviews on the topic of ED intervention programmes (published up until December 2020) as well as references in identified studies were searched for further relevant publications.

### Eligibility criteria

Included were publications fulfilling the following criteria: (i) published in a peer-reviewed journal; (ii) publication language is English or German; (iii) longitudinal, primary study type, i.e. original data of at least one study arm and two assessment time points; cross-sectional studies were also included if at least one RE-AIM dimension was reported in the article (e.g. reach for an Internet-based programme); (iv) the programme covers universal, selected or indicated prevention; (v) the programme aims to prevent EDs or reduce risk factors for EDs, defined by fulfilling one of the following criteria: (a) is declared as ED prevention programme by study authors, (b) aims at reducing body image concerns or body dissatisfaction or (c) promotes balanced eating habits (e.g. excluding programmes with the primary goal of weight loss or focusing solely on caloric intake, or healthy/unhealthy food intake); (vi) the programme is fully or partly technology-based and delivered via computer, tablet or smartphone including blended interventions combining face-to-face and technological delivery modes; and (vii) the programme is targeted at the prevention of EDs in adults. We also included studies with samples aged younger than 18 if parts of the sample were older than 18 and if the intervention was not limited to high school students only.

We excluded studies reporting on interventions for treatment of individuals with fully diagnosed EDs. Publications reporting on both prevention and treatment were included if the study sample consisted of less than 50% of individuals with a fully diagnosed ED or if they reported separately on prevention effects. We included non-randomized controlled trial (RCT) publications as well as RCTs (see eligibility criterion 3) to be able to cumulate information from a great variety of study types. Non-RCT publications yield valuable information on how an intervention works in less controlled conditions or routine practice and on external validity indicators, in addition to RCTs, which focus on efficacy under highly controlled conditions and mainly yield information on internal validity.

### Study selection

After removing duplicate articles and non-journal publications (books, book chapters, theses, conference abstracts), all remaining abstracts were screened for eligibility. Then, full-texts of relevant publications were obtained and assessed for inclusion. Both steps were conducted by at least two researchers independently (B.N., M.Z. and S.K.). In case of discrepancies, a senior researcher (K.W.) was consulted to reach consensus.

### Adapting the RE-AIM framework for a review on Internet-based interventions

The RE-AIM framework defines reach as ‘the absolute number, proportion, and representativeness of individuals’ willing to participate in a programme and reasons for non-participation.^18^ For this review, we considered reach in several ways to accommodate the Internet-based enrolment: we defined ‘participation rates’ as (i) the number of screening participants divided by the number of approached individuals, (ii) the number of consenting participants divided by the number of eligible individuals, (iii) the number of randomized participants divided by the number of eligible individuals or (iv) the number of participants who provided baseline data divided by the number of eligible/randomized participants. We also included an initial ‘uptake rate’ that includes the number of people who accessed the intervention at least once divided by the number of participants who were randomized and provided access. Effectiveness in the RE-AIM model describes the impact of an intervention on important outcomes, including potential negative effects, quality of life and economic outcomes.^18^ Adoption is defined as the absolute number, proportion and representativeness of settings and intervention delivery agents who are willing to initiate a programme.^18^ Implementation at the individual level refers to participants’ use of the intervention and implementation strategies. At the setting level, implementation refers to the delivery agents’ fidelity to the intervention’s protocol.^18^ Maintenance describes both the extent to which participants behaviour change is maintained (defined as ≥6 months for this review) after the intervention (individual maintenance) and the extent to which the programme is sustained and becomes institutionalized or part of routine organizational practices (setting-level maintenance).^18^

Data from eligible articles were extracted using a previously validated coding sheet for RE-AIM reviews.^19^^,^^20^ As not all items and definitions of the original RE-AIM model fit the conceptualization and implementation of Internet-based interventions, the coding sheet was adapted and amended to account for the specific needs of this review. For example, Internet-based interventions can be offered outside of specific settings, which was taken into account by coding setting-related information only if interventions were indeed setting-based. All extracted RE-AIM items (subsequently called indicators) as well as explanations for modified and additional indicators are provided in Supplementary table S2. All adaptations were discussed with an RE-AIM expert (L.M.K.) and members of the ICare research consortium who are all experienced in delivering Internet-based interventions.

### Data extraction and scoring

Overall, 43 RE-AIM indicators were extracted: 10 for reach, 7 for efficacy/effectiveness, 12 for adoption, 9 for implementation and 5 for maintenance. The coders (B.N. and M.Z. or S.K.) independently coded ‘yes’ vs. ‘no’ for the presence or absence of each indicator and if present extracted the respective information. The coders agreed in 91.1% (Cohens’ *κ*: 0.88) and 90.4% (Cohens’ *κ*: 0.81) of coding decisions (indicator reported vs. not reported) across all RE-AIM indicators, suggesting good inter-rater reliability. Discrepancies were resolved by discussion with a third independent researcher (K.W. or L.M.K.). The percentage of reported indicators in each of the five RE-AIM dimensions was calculated per study. We calculated exclusion and participation rates of participants when sufficient data were available in the respective publication. Analyses also included summative reporting rates (percentage) for each RE-AIM indicator and each dimension across all studies. Additionally, fostering and hindering factors for reach, adoption, implementation and maintenance were extracted by two independent researchers (B.N. and M.Z. or S.K.) when mentioned in the articles. For these factors, the coders collected qualitative information reported in the publications that they regarded as relevant. Common themes and categories were identified from the extracted information in the style of a thematic analysis approach.^21^ Discrepancies were resolved by discussion between the coders.

## Results

### Study selection

**Figure 1:**
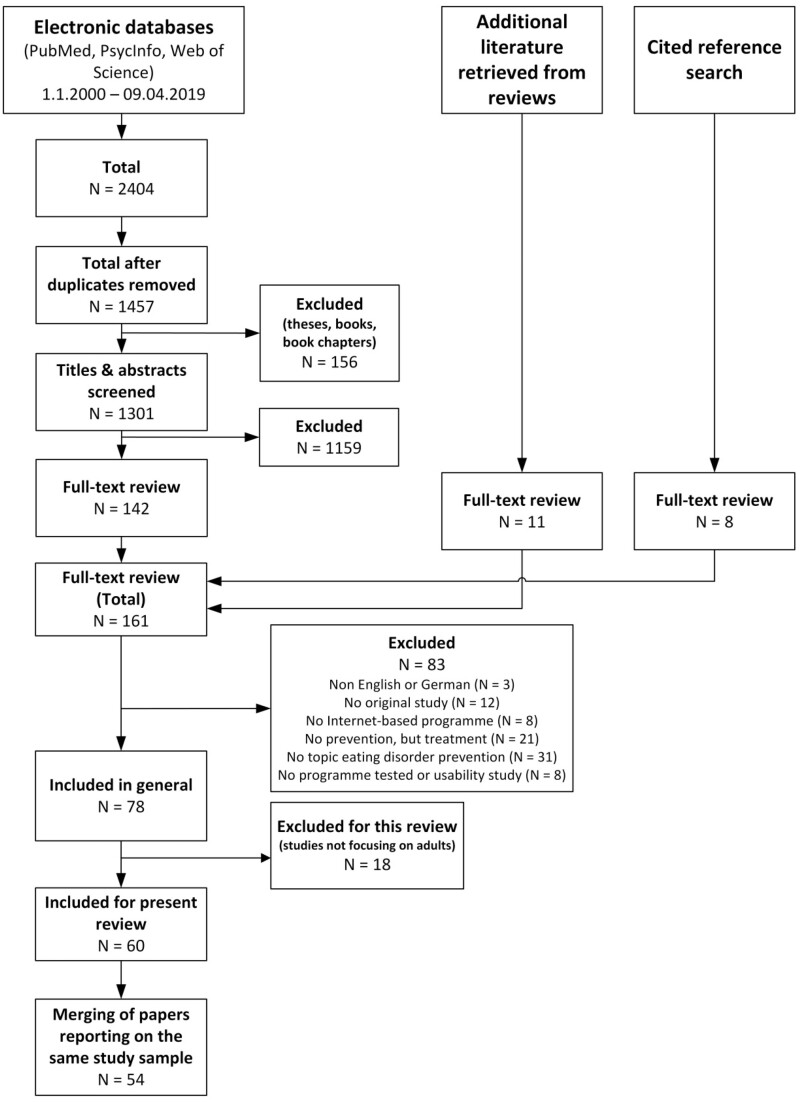
Flow diagram of studies included in the review

The process of study selection and reasons for exclusion are presented in figure 1. The database search yielded 928 records from PubMed, 671 records from PsycINFO and 805 records from Web of Science, totalling 2404 records; 1301 records remained after exclusion of duplicates and excluding non-journal articles, which were then screened for eligibility based on title and abstract resulting in 142 articles considered for full text review. An additional 11 full-texts were retrieved from published reviews and 8 full-texts by screening reference lists of included studies. A total of 161 full-texts were checked for eligibility. Of those, 83 were excluded for not fulfilling inclusion criteria and 18 focused on adolescents only and were excluded for the purpose of the current review. Twelve of the included articles reported on the same study sample and were merged. An additional four articles reported on the same study but analysed different subsamples and therefore were included as separate studies. Overall, a total of 60 articles representing 54 individual studies were included.

### Study and intervention characteristics

Study and intervention characteristics of all included studies are shown in Supplementary table S3. The majority of studies was conducted in the USA (*n* = 31; 57%), followed by Germany (*n* = 9; 16.7%) and Australia (*n* = 6; 11%). In total, studies from 12 different countries were included. Most studies (*n* = 37; 69%) reported on RCTs. The remaining studies were conducted as uncontrolled and/or non-randomized pilot studies, feasibility/acceptability studies, observational study, cross-sectional studies (e.g. reporting on reach), feasibility or dissemination^22^/implementation^23^ studies. Seventeen studies reported on a version of the *StudentBodies*/*StayingFit*/*Image and Mood*/*Healthy Body Image*-Suite, five studies on a (translated) version of *ES[S]PRIT*/*ProYouth*, three studies on *Expand your Horizon*, three on *Food, Mood and Attitude*, two on *Set Your Body Free*, two on the *eBody Project*, two on the intervention used in the Young Adults Eating and Active for Health-Project and two studies reported on the same version of meditation exercises. Twenty-one studies focused on universal, 17 on selected and 27 on indicated prevention programmes whereby 10 of those covered 2 or 3 prevention levels, e.g. by recruiting participants at different levels of risk. Five studies reported using a tailored approach by offering different components or interventions based on participants’ risk status (segmented population), three studies offered tailoring by letting participants choose intervention components based on their preferences and two studies used stage-based intervention components adapted to participants’ status of change.

### RE-AIM dimensions

The reporting rates for each single RE-AIM indicator across studies are presented in table 1. The total reporting rate across all studies and all RE-AIM dimensions was 46%, with the highest reporting rate in the reach dimension (63%), followed by implementation (57%), effectiveness (54%), adoption (24%) and maintenance (21%). Supplementary table S3 provides the reporting rates of the RE-AIM dimensions per study and Supplementary table S4 provides the reporting status for each RE-AIM indicator per study. An overview of fostering and hindering factors is provided in Supplementary table S5.

**Table 1:** Reporting rates for RE-AIM indicators across studies (*N* = 54)

RE-AIM indicator	Reporting rate (%)	RE-AIM indicator	Reporting rate (%)
Reach (total)	62.6	A5. Characteristics of approached setting (*n* = 49)^a^	30.6
R1. Method to identify target population	72.2	A6. Characteristics of non-approached settings (*n* = 49)^a^	0.0
R2. Inclusion/Exclusion criteria	94.4	A7. Representativeness of participating settings (*n* = 49)^a^	2.0
R3. Exclusion rate	50.0	A8. Reasons for declining of settings (*n* = 49)^a^	2.0
R4. Sample size	100	A9. Method to identify delivery agent (*n* = 21)^b^	4.8
R5. Participation rate/uptake rate	64.8	A10. Description of staff delivering intervention (*n* = 21)^b^	57.1
R6. Characteristics of participants	100	A11. Level of expertise of delivery agent (*n* = 21)^b^	42.9
R7. Characteristics of non-participants	1.9	A12. Start-up costs	0.0
R8. Representativeness of participants	29.6	Implementation (total)	57.0
R9. Reasons for declining participation	16.7	I1. Format of intervention	98.1
R10. Recruitment strategies	96.3	I2. Frequency and intensity of intervention	88.9
Efficacy/effectiveness (total)	54.2	I3. Level/Type of staff support needed	96.0
E1. Measures and results for post-intervention assessment	85.2	I4. Electronic devices used	40.7
E2. Intention-to-treat analysis utilized	51.9	I5. Extent to which intervention was delivered as intended	74.1
E3. Imputation procedure	46.3	I6. Consistency of intervention delivery	14.8
E4. Quality of Life measure included	14.8	I7. Costs of delivery	7.4
E5. Measure of satisfaction with/acceptability of programme	35.2	I8. Incentives used	55.6
E6. Effects at follow-up	59.3	I9. Data protection measures	37.0
E7. Attrition	87.0	Maintenance (total)	21.5
Adoption (total)	24.2	M1. Assessed outcomes ≥6 months (individual level)	31.5
A1. Type(s) of included settings (*n* = 49)^a^	95.9	M2. Drop-out rate to last follow-up (*n* = 17)	100
A2. Geographical characteristics of setting (*n* = 49)^a^	69.4	M3. Current status of programme (setting level)	20.4
A3. Inclusion and exclusion criteria for settings (*n* = 49)^a^	2.0	M4. Adaptations made	5.6
A4. Adoption rate (*n* = 49)^a^	4.1	M5. Costs of maintenance	3.7

a Forty-nine studies utilized a setting for recruitment and/or intervention delivery, while five studies reported an online setting only and indicators were not applicable in this case.

b Twenty-one studies utilized a delivery agent or gatekeeper for intervention delivery, while 33 did not and indicators were not applicable in this case.

### Reach

Most studies (94.4%) reported on *inclusion and/or exclusion criteria*. Inclusion criteria mostly referred to female gender, age, self-reported body dissatisfaction or the wish to improve body image, symptoms of EDs and varying BMI ranges. Current or past full-syndrome EDs, current or past ED treatment, bingeing/purging behaviours, low BMI, pregnancy, suicidal ideation and psychological disorders were the most commonly reported exclusion criteria. Studies that did not report inclusion/exclusion criteria in fact did not exclude participants.^24–26^

Of the total studies, 72.2% reported on *methods used to identify the target population*. Most studies (*n* = 36) used an online, in-person or telephone screening procedure, while three studies had participants confirm their body image concerns as a method of verification. Half of the studies (50%) provided data that allowed for calculation of an *exclusion rate*. Exclusion rates ranged between 0% and 93.0% (median: 25%), with studies reporting on selected or indicated prevention programmes producing the highest exclusion rates. All of the studies provided a *sample size*, which ranged from 4 to 4051 (median: 139; 25th quantile: 64; 75th quantile: 379). Thirty-five (64.8%) of the studies provided data that allowed for calculation of a *participation rate*. Of those, four studies reported the number of participants who took part in the study’s screening compared with the number of approached individuals (participation rates: 2.5% to 18.4%; median: 8.1%). Another nine studies contrasted the number of participants who registered/agreed to participate to those who were eligible ranging from 12% to 99.5% (median: 41%). Fifteen studies reported the rate of the number of randomized participants compared with those eligible and/or agreeing to take part (but ultimately did not participate or respond), ranging from 20.8% to 94.3% (median: 58.5%). Four studies compared the number of participants who provided baseline data with the number of participants who were eligible and/or randomized to a condition, resulting in participation rates from 31.9% to 100% (median: 87.1%). Another three studies contrasted the number of participants who accessed the intervention at least once to the number of participants who were randomized and provided access to the intervention (uptake rate: range 57.7% to 90.3%; median: 85%).

All of the studies described core *characteristics of participants*. More than half of the studies (64.8%) had female-only samples. One study did not report on the percentage of males/females. The remaining 18 studies reported predominantly female samples, with the percentage of females ranging from 32% to 95.6% (median: 74.7%). Mean age of participants (reported by 92.6% of studies) ranged from 15.7 years to 53 years (median: 21.3 years), with three studies approaching both adolescent and adult populations.^22^^,^^27^^,^^28^ In nine studies, the mean age ranged between 30 years and 50 years and two studies reported a mean age of more than 50 years for their respective sample. Thirty-six studies reported data on the ethnicity and/or race of participants. In those, the percentage of non-Caucasian participants ranged from 5% to 100% (median: 39.9%), with two studies exclusively targeting a Latina population.^29^^,^^30^ One study provided some information about *characteristics of non-participants.*^31^ In this study, eligible individuals who did not register for the programme had lower BMI values, lower weight and shape concern scores and lower severity scores for bulimic core symptoms than eligible individuals who had registered for the programme. Sixteen studies (29.6%) reported on the *representativeness of participants*. In five of those studies, the study samples had similar scores in measures of body dissatisfaction or ED-scores compared with either a non-clinical sample or help-seeking individuals^32^^,^^33^ or were regarded as diverse in their ethnic background.^34–36^ Conversely, in 10 studies, the study sample differed in measures of body dissatisfaction,^37–39^ ED (risk) scores,^23^^,^^31^^,^^40^ educational level,^37^^,^^41^^,^^42^, sex^37^ or ethnic background^37^^,^^41^ compared with reference samples (i.e. non-clinical community or college samples, healthy norm samples or census data). Two studies reported mixed results regarding representativeness, with only some measures comparable to the control sample.^43^^,^^44^*Reasons for declining participation* (reporting rate: 16.7%) were lack of time/scheduling conflicts, lack of interest or withdrawal of consent with no reason specified.

Most studies (96.3%) reported on *recruitment strategies*, with the majority of studies (*n* = 26) utilizing both online and offline strategies, most commonly via a study webpage, mass e-mails, social media, flyers and announcements in lectures. Eighteen studies reported offline only recruitment, five studies used online only methods, while three studies did not specify their means of recruitment.

Anonymous, low-threshold access of Internet-based interventions^24^^,^^29–32^^,^^39^^,^^45^ as well as flexible use independent of time and geographical location^24^^,^^26^^,^^38^^,^^46^ were repeatedly mentioned as *fostering factors for reach*. Less strict exclusion criteria also extended reach in some studies.^47^^,^^48^ Offering the intervention at no costs for participants was mentioned as another fostering factor.^31^^,^^49^ Embedding recruitment in an educational setting (university campus^23^, or vocational training^50^) was also seen as beneficial for reach.

As *hindering factors for reach*, several authors mentioned their recruitment strategies as possibly unsuitable to reach a diverse population or the population most in need for the intervention.^37^^,^^51^^,^^52^ Computer/technology literacy might have posed a barrier for some.^51^^,^^53^ Having the interventions available for one operating system, only could have influenced reach negatively, as mentioned in one study.^23^

### Effectiveness

Most studies (85.2%, *n* = 46) reported *measures and results for post-intervention assessments*. Of those, 28 (60.9%) utilized an *intention-to-treat analysis* and 25 (54.3%) reported on the *imputation procedure* that was used. The most frequently reported measures included assessments of weight and shape concerns and body image (*n* = 31), ED symptoms (*n* = 30), BMI or body measurements (*n* = 17) and measures of depression (*n* = 13), while 14.8% of studies included a measure for *quality of life* or clinical impairment. *Follow-up effects* were reported by 32 studies (59.3%), with half (*n* = 16) of those reporting on short-term follow-ups (less than 6 months) only. Nineteen studies (35.2%) reported on qualitative and/or quantitative measures of *participants’ satisfaction with/acceptability of the programme*. Assessment *attrition* (non-completion of post-assessments) was reported by most studies (87.0%) and ranged from 0% to 81.0% (median: 13%; 25th quantile: 8%; 75th quantile: 36%).

### Adoption

In most studies (92.2%), recruitment for the trials was *setting-based*, while intervention delivery was mostly independent of setting. Recruitment settings included mainly university and college campuses, but also student counselling centres, employer or health insurance plans and a hospital. Five studies conducted recruitment and intervention delivery online and did not provide further information on the setting. Of studies that used a setting for recruitment and/or intervention delivery (*n* = 49), 34 specified the *geographic area* (e.g. cities, regions, or whole countries) the study was conducted in. Fifteen studies provided further *characteristics of the approached setting*, which were predominantly described as either public or private universities and rural or urban location of the university. *Characteristics of non-approached settings* were described in none of the studies. One study reported on the *representativeness of the participating setting* by describing its limited generalizability to other regions and populations not approached in this study.^53^

One study specified *inclusion criteria for the setting*, which was defined as having an on-campus clinical representative for the intervention, and a *reason for declining a setting*.^23^ The same study had an *adoption rate* of 62%, reporting the number of participating universities in proportion to those approached.^23^ Twelve studies included a *description of staff delivering the intervention*. They were mostly described as psychology (graduate) students, clinical psychologists or student counsellors. Regarding the *level of expertise of delivery staff*, nine studies employed bachelor’s students, graduate students in clinical psychology, clinical psychologists (with some specialized in EDs and body image) and three of those studies reported providing training for the delivery staff beforehand. None of the studies reported on *start-up costs* for setting up the intervention.

One study considered the Internet-based format of the intervention as *fostering for adoption*, as it solves the problem of having to identify university clinicians to deliver the intervention.^54^ College infrastructure was recognized as fostering for adoption of prevention programmes^55^ as well as implementing mandatory ED screenings in a college setting in future dissemination efforts.^23^ However, potential costs for screening and treatment at campuses were mentioned as possible *hindering factors*.^23^ Having participants randomized to either online or offline groups in research settings and perhaps not meeting participant’s preferences with this procedure was additionally mentioned as potentially hindering for adoption.^56^

### Implementation

The *format of intervention* was reported by all but one study (98.1% reporting rate). Most studies (*n* = 45) provided the intervention in a web-based only format, with seven studies using a blended format combining face-to-face and online sessions. Three studies offered the intervention via a mobile app with two of them providing additional face-to-face sessions. Another study switched the format from web-based to mobile app during the course of the trial.^23^ Three studies offered the intervention (‘Food, Mood and Attitude’) mainly in CD-ROM format,^29^^,^^30^^,^^57^ and one intervention consisted of downloadable audio podcasts that were offered online.^58^

The *electronic devices* intended to access the intervention (reported in 40.7% of the studies) were mostly personal or lab computers (*n* = 16), while four studies used smartphones only and one study explicitly developed the intervention for computer and smartphone use.

Most studies (96.3%) reported information on *the frequency and intensity of the intervention*. One study allowed for a flexible use of modules for a period of 8 months,^23^ while the Es[s]prit/ProYouth/Appetite for Life intervention was offered for flexible use without a recommendation or restriction of duration.^22^^,^^27^^,^^28^^,^^31^^,^^40^ Of the remaining studies, 43 provided information on the intended duration of the intervention, which ranged from 4 days to 14 weeks (median: 8 weeks; 25th quantile: 3 weeks; 75th quantile: 9.5 weeks). Twenty-four studies reported the intended weekly input time, ranging from 12 minutes to 140 minutes (median: 60; 25th quantile: 38.8; 75th quantile: 90 minutes).

Three of four studies (74.1%) reported information about the *extent to which intervention was delivered as intended*, with the majority reporting on participants’ adherence to the intervention. Studies that evaluated a similar prevention programme from the same ‘family’ of interventions used common or similar measures for adherence more often, otherwise adherence measures differed greatly between studies, reporting e.g. the percentage of completed modules of the intervention, mean/median number of modules completed, mean/median number of days/weeks/months of use or mean/median total duration of activity. In seven studies, a percentage of full programme completion was reported, ranging from 1.4% to 89% of participants who completed all sessions of the intervention (median: 49.3%; 25th quantile: 27.4%; 75th quantile: 76.1%). Three studies reported the percentage of participants continuing on past the first session, ranging from 12.1% to 60.4% (median: 43.2%; 25th quantile: 24.3%; 75th quantile: 58.6%).^23^^,^^33^^,^^48^ Apart from these measures of adherence, four studies reported measures of participants’ compliance with the task by checking the content of writing exercises^59–62^ and another study reported staff adherence to the manual.^33^

Most studies (88.9%) reported the *level and type of staff support* needed for intervention delivery. Tasks included moderating asynchronous group discussions (reported by 17 studies), monitoring user progress/sending reminders (*n* = 11), moderating synchronous group chat sessions (*n* = 8), providing individual feedback to participants (*n* = 7), individual chat sessions (*n* = 6), providing a face-to-face introduction to the intervention (*n* = 6), offering tech (*n* = 3) or telephone support (*n* = 1), sending out summaries and readings (*n* = 2) and reviewing transcripts of chat sessions or homework (*n* = 3). One study reported delivering a guided intervention but did not specify guidance activities. In 12 studies, interventions were delivered without any guidance.

Few studies (14.8%) provided information on *consistency of intervention delivery*. In three studies, an instruction manual was provided to staff to ensure standardized delivery, while another study used the same facilitator for each chat group to provide comparable input. One study switched the delivery mode from web-based to mobile-based. Three studies mentioned the flexible use of the intervention and two of those compared the user behaviour of subgroups of participants.

More than half of the studies (55.6%) reported on *incentives* for participants including vouchers, cash or course credit to compensate for completion of assessments or intervention components.

Information on *data protection measures* was reported by 37.0% of the studies. These measures usually included anonymous usernames, password-protected access and/or secured servers. Two studies provided specific information on data security measures such as encryption of data, security and privacy training for staff and citing security standards that were met.^34^^,^^45^

Four studies (7.4%) reported on *costs of delivery*, with two of those providing information on time expenditure for staff,^53^^,^^63^ which was 1–2 hours per week for moderating group discussions. Two calculated a rate of costs per participant of 15€ per year (evaluation period from November 2011 to February 2013), plus unquantified costs for setting up implementation,^22^ and 26$ total cost per participant (data collection between September 2009 and April 2012), respectively.^36^

Regarding *fostering factors for implementation*, low implementation costs were mentioned by a number of authors.^36^^,^^37^^,^^57^^,^^59^^,^^64^ Several researchers emphasized intervention delivery as feasible^22^^,^^37^^,^^46^^,^^59^^,^^64^ and time-saving.^62^^,^^65^ Flexible, self-directed use^22^^,^^31^^,^^34^^,^^38^^,^^56^^,^^57^^,^^62^ and providing access to Internet/computers^66^ were mentioned as fostering as well. Discussion groups,^26^^,^^38^^,^^51^^,^^63^ anonymous participation^22^^,^^32^ and electronic reminders^36^^,^^48^ were considered as beneficial for engagement of participants. Implementation at a vocational training site might have helped to integrate the intervention into daily routine in one study.^50^ Combining screening and intervention was also seen as beneficial for implementation.^45^^,^^65^ Self-selected samples^67^^,^^68^ and high motivation of participants^53^ were considered as possibly increasing engagement.

*Hindering factors* for implementation included technical problems during the study,^32^^,^^34^^,^^41^^,^^60^^,^^68^^,^^69^ usability issues^33^^,^^41^^,^^48^^,^^63^^,^^65^ and restricted access to computers.^50^ Varying computer competency of participants,^32^^,^^53^^,^^68–70^ delay between screening and start of the intervention,^34^^,^^65^ lack of personal interaction^32^^,^^48^^,^^56^^,^^71^ and privacy concerns^66^^,^^69^ were mentioned as hindering for participants’ engagement. Authors mentioned time constraints of participants,^34^^,^^38^^,^^41^^,^^49^ motivational problems,^29^^,^^33^^,^^41^^,^^72^^,^^73^ not meeting participants needs and interests,^34^^,^^41^^,^^70^^,^^74^ health problems,^41^^,^^70^ family issues,^70^ an all-female staff in a mixed-gender intervention^22^ and lower commitment due to anonymity^31^ as detrimental to adherence. One study had to be discontinued due to participants’ withdrawals and staffing challenges.^64^

### Maintenance

Of the studies, 31.5% assessed *individual participant follow-up outcomes 6 or more months after the intervention completion*, with follow-up periods stretching up to 3 years. All of those reported *drop-out rates* up to the last follow-up assessment, which ranged from 4.7% to 81.8% (median: 21%; 25th quantile: 15%; 75th quantile: 36%). Eleven studies (20.4%) provided sustainability information about the *current status of the programme*. Three of the interventions were still accessible at the time of publication,^22^^,^^49^^,^^58^ six studies reported plans for further adaptations and studies^40^^,^^44^^,^^45^^,^^54^^,^^72^^,^^74^ and two reported current evaluation of the (adapted) interventions.^34^^,^^63^

In two studies (3.7%), *costs for maintenance* were mentioned, with one study citing the implementation costs as applicable for maintenance, i.e. 15€ per participant plus unquantified dissemination costs,^22^ and another relating costs to the number needed to treat, resulting in costs of $130 to $390 to prevent one case.^36^

High potential to feasibly disseminate an existing intervention to further settings, e.g. health services or educational courses, was deemed fostering for the maintenance of programmes.^24^^,^^26^^,^^30^^,^^37^^,^^39^^,^^45^^,^^46^^,^^52^^,^^69^^,^^75^ Sponsoring,^23^ high stakeholder involvement^63^ and promotion of programmes^22^ were mentioned as facilitating factors for maintenance. Several authors mentioned specific features such as automated components and procedures,^23^^,^^48^ coaches dashboards^34^ and manuals^51^^,^^63^ as beneficial for scalability and real-world application of interventions. *Hindering factors* concerned limited funding and staff resources,^23^ especially in research-funded interventions.^63^

## Discussion

The aim of this review was to summarize existing research on reach, effectiveness, adoption, implementation and maintenance of ED prevention programmes for adults to provide insights informing the future implementation and dissemination of interventions for adults. The present review was conducted in parallel with a review on Internet-based ED prevention programmes for adolescents^16^ to provide an overview spanning different age groups from adolescents to adults. Our analysis revealed that the indicator reporting rates of the dimensions *reach*, *effectiveness* and *implementation* amounted to more than 50%, while the rates for *adoption* and *maintenance* were below a total of 25%. These reporting rates were mostly comparable (i.e. within a 10% range) with the RE-AIM review on programmes for adolescents, except for adoption attaining a higher rate in the adolescents’ review.^16^

### Reach

Inclusion/exclusion criteria, sample size, sample characteristics and recruitment strategies were the best-reported indicators for reach with reporting rates of at least 90%. For this review, we calculated participation rates based on a number of varying indicators, i.e. approached or eligible individuals, screened, registered, consented or randomized participants, or participants with baseline data. Since at least the number of participants randomly assigned, receiving intended treatment and providing baseline data are expected to be included in a CONSORT flow chart figure,^76^ 64.8% of studies providing data for calculation of a participation or uptake rate constitute a low percentage, given the fact that 69% of the studies were RCTs but were comparable to ED prevention interventions for adolescents^16^ or reviews on other domains, such as physical activity promotion interventions.^77^^,^^78^ Future publications could provide valuable information by adhering to the CONSORT recommendations,^76^ even when reporting a non-RCT study.

Reporting rates for representativeness of participants were better than in the review on ED prevention interventions for adolescents^16^ (30% vs. 9%), but still sparse. The majority of studies providing a comparison of participants vs. non-participants suggested that samples were not representative regarding the chosen measures. Considering the paucity and lack of comparability of these data, it is unknown whether the evaluated interventions would still reach comparable effects within broader dissemination contexts. Even with some studies reporting samples’ characteristics representative of the general or target population, a more detailed description of recruitment strategies to better comprehend and replicate sample representativeness is needed. In turn, one study provided an example of a strategy to reach a more balanced distribution of sex in their sample by undertaking measures directed at males.^43^ University samples might cover the intended target group of young women to some extent but to reach participants irrespective of educational level and to gain larger public health impact, community samples are needed.^79^ In general, to be able to estimate effects in larger-scale dissemination, the field would benefit from adding information on the underlying target populations and then comparing their characteristics to the sample actually reached in the study.^18^ Further, Glasgow et al.^80^ suggest an expanded version of the CONSORT flow diagram figure, which includes a report of key differences of participating and non-participating individuals.

### Effectiveness

Most studies reported on efficacy by providing body image-related or ED-related outcomes. With the majority of studies conducting RCTs, the current review revealed that the available research focuses on efficacy rather than real-world effectiveness. The utilization of quality of life-related outcomes, which would allow for cross-study comparisons, was low, but higher than in the review focusing on adolescent programmes (14.8% vs. 0%).^16^ Similar to a systematic review on ED prevention at universities, measures for quality of life were heterogeneous, showing the need for a more consistent approach to assess functioning effects of ED prevention interventions.^9^ Measurements of user’s acceptability towards the online programme differed greatly between studies as well, calling for standardized measures to establish comparability between studies. Almost all studies reporting outcome measures also reported on attrition, with highly varying rates. Higher attrition rates might reflect a less controlled study design and could therefore be expected in a more real-world dissemination practice.^79^ This raises the issue of dealing with potentially rising attrition rates when interventions are disseminated or implemented in real-world settings.^81^ However, a recent meta-analytic review, reporting on RTCs only, found high heterogeneity of reported attrition as well, while attrition was higher in newer trials (post-2015),^10^ indicating that attrition needs to be considered in all study types and settings.

### Adoption

Reporting rates for adoption indicators were generally lower in adult studies compared with studies on online programmes targeting adolescents (total reporting rates of 24.2% vs. 34.7%, respectively).^16^ This may reflect the difference in how ED prevention programmes are implemented in the adolescent or adult population: while many online preventive interventions for adolescents are delivered in the school setting,^16^ setting-based approaches are rarer in the delivery of adult programmes. Building on the existing literature on lifestyle behaviour interventions indicating that setting-based interventions generally produce better outcomes regarding effectiveness, reach and use of interventions,^82^ future research on Internet-based ED prevention programmes for adults may focus on implementation in settings that provide access to the respective target audience. For example, college infrastructure could provide a feasible setting,^23^^,^^55^ but at the same time, reach of a non-college population, e.g. via workplaces or healthcare settings, has to be considered. While the majority of studies reported some information about the geographic region of recruitment/intervention delivery, further setting-related information, including adoption rates, characteristics of (non-)approached settings or costs of setting up the intervention was reported very sparsely (<5%). The majority of studies provided a description of staff, if applicable, but most did not include selection criteria for staff or delivery agents. We therefore cannot conclude whether staff selection was a matter of convenience or cost-efficiency, e.g. by employing psychology (graduate) students, or whether the same level of expertise would be needed/sufficient in a real-world adoption as well. To improve reporting of factors relevant to external validity, an expanded CONSORT figure has been proposed that includes the number of potential participating settings and staff, number of included, participating, and declined settings and staff, and their key differences.^80^

### Implementation

Intervention features (staff support, format, intensity and frequency of the intervention) were well reported in the existing literature. Most studies were fairly precise in describing the amount of time and work that was expected from participants. The majority of studies reported on individual’s adherence measures but used very heterogeneous measures. Use of heterogeneous adherence measures across studies is commonly reported for Internet-based interventions for mental health^10^^,^^83^^,^^84^ but limits comparability of interventions and generalizability of findings related to adherence. Initial use and engaging more people in interventions might be some of the most important steps to improve public health impact of ED prevention.^14^^,^^85^ To understand the reasons for intervention dropout to prevent it,^85^ future publications could report the events (e.g. number of sessions), characteristics of sessions or session content at which participants drop out. Reviews on adherence reporting in Internet-based interventions propose reporting multiple adherence measures and inclusion of common measures across trials,^83^^,^^84^ which could be applied to the field of Internet-based ED prevention.

Reporting of implementation costs was sparse (less than 10% of studies). Although several authors described intervention delivery as affordable or as less costly than face-to-face alternatives, few provided data that underlined or validated statements about costs, a gap which is commonly reported in other RE-AIM reviews as well.^16^^,^^77^^,^^86^ Moderation of group discussions and guidance of interventions were mentioned as a significant expense. Considering that some amount of guidance seems warranted in indicated prevention programmes and guidance has been found to be positively associated with better outcomes,^87^ precise reporting of staff time and costs is key to inform future dissemination efforts. Indeed, cost of moderation and guidance is seen as one barrier for long-term dissemination,^79^ but ultimately, ED prevention has previously been shown to be less costly than waiting and providing treatment only to ED cases.^88^ Further studies are needed to find out which interventions and which levels of prevention can reach cost-effectiveness.

Given the importance of data security and privacy in E-health interventions,^89^ standardized reporting of key aspects of data security (e.g. encrypted connections, password protection or security protocols and policies for staff) is warranted. Less than 40% of studies included in the present review have reported any data protection measure, highlighting the potential for improvement.

### Maintenance

Similar to online programmes for adolescents,^16^ data on the sustainability of effects and programmes are scarce for online ED prevention interventions for adults (total reporting rates of 18.2% and 21.5%, respectively). Indeed, very few studies reported follow-up data beyond one year, limiting the evidence for a sustained intervention effect for participants. While there is promising evidence that Internet-based ED prevention programmes can be efficacious at participant level in the long-term,^6^^,^^7^^,^^90^ very few studies provide information on programme sustainability on a setting level past their funding period. This might be due to funding ending, which thus limits the collection of data on sustainability.^80^ Authors are encouraged to provide the number and information on settings in which the intervention is or is not continued in an expanded CONSORT figure.^80^ However, a factor limiting programme sustainability from the start might be the design process of interventions, as they are often conceptualized in research settings without scale-up in mind and without consideration of requirements, processes and limitations in routine care settings and the needs of all involved stakeholders.^91^ Providing the reasons why specific settings or delivery agents declined participation would help programme developers to plan scale-up from the beginning and to develop strategies to increase adoption.^92^

Overall, this review gathered published information on RE-AIM dimensions for a great variety of Internet-based ED prevention programmes with diverse theoretical backgrounds. The review reveals reporting gaps of indicators relevant to dissemination and implementation in real-world settings, such as careful evaluation of reached individuals in relation to the target groups, characteristics and representativeness of settings, data to estimate cost and cost-effectiveness and data security. We would like to encourage the use of the expanded CONSORT figure as proposed by Glasgow,^80^ which includes several RE-AIM indicators, e.g. characteristics of non-participants or excluded settings, or status of the intervention past the initial funding period.

### Limitations

The findings of this review have to be considered with a few limitations in mind. We included papers concerning different types of study designs, ranging from small pilot studies to dissemination trials. The majority of included papers reported on RCTs, which do not necessarily demand reports on dissemination-related indicators. This might reduce reporting rates for measures, especially of external validity. Furthermore, we did not search for grey literature or unpublished data, so our review only includes information from papers that were identified by our inclusion criteria. Lastly, some RE-AIM indicators such as *types of settings*, *adoption rate of settings* or *consistency of intervention delivery* were difficult to apply, e.g. when settings were only (partly) used for recruitment, and not intervention delivery.

## Conclusions

Internet-based ED prevention interventions are regarded as scalable and feasible for dissemination. However, few studies have shown successful implementation outside a controlled research setting so far. Improved reporting of RE-AIM indicators, particularly in the dimensions adoption, implementation and maintenance, could help inform possible strategies to move the field towards maintained implementation. Common measures for individual adherence and quality of life would improve comparability across interventions and studies.

## Supplementary data

Supplementary data are available at *EURPUB* online.

## Funding

This project has received funding from the European Union’s Horizon 2020 research and innovation programme under grant agreement No 634757. 

*Conflicts of interest*: None declared.

**Key points**

- The available literature on Internet-based ED prevention provides little data on real-world effectiveness, dissemination and sustainability of interventions.
- Study participants are rarely fully representative of target groups.
- Measures of intervention adherence and programme acceptability diverge widely calling for more harmonized approaches to establish comparability between studies.
- While recruitment in most studies was setting-based, implementation of prevention programmes was often detached from the setting.
- Reporting of RE-AIM indicators may facilitate strategies and design decisions that consider dissemination and scale-up from the beginning.

## Supplementary Material

ckab044_Supplementary_Material
