# Supplementary material for: A systematic review of reach, adoption, implementation and maintenance of Internet-based interventions to prevent eating disorders in adults
Source: Eur J Public Health. 2021 Jul 7;31(Suppl 1):i29–37. doi: 10.1093/eurpub/ckab044 (PMC8266539; doi:10.1093/eurpub/ckab044)
Supplement: ckab044_Supplementary_Material [file ckab044_supplementary_material.pdf]

Electronic supplementary material

Supplement to:

A systematic review of reach, adoption, implementation and maintenance of Internet-based interventions to prevent eating disorders in adults

European Journal of Public Health

Barbara Nacke, Michael Zeiler, Stefanie Kuso, Lisa Klesges, Corinna Jacobi, Karin Waldherr

Corresponding author:
Barbara Nacke
Technische Universität Dresden, Institute of Clinical Psychology and Psychotherapy, Chair of Clinical Psychology and E-Mental-Health, 01062 Dresden, Germany
E-mail: barbara.nacke@tu-dresden.de

Table of content

Table S1. Search syntax used for literature search in PubMed, PsycINFO and Web of Science 2
Table S2. Adapted RE-AIM coding sheet used for the present systematic review 3
Table S3. Study and intervention characteristics of included studies and RE-AIM reporting rates of included studies 6
Table S4. Reporting status of RE-AIM indicators for each included study 11
Table S5. Overview of fostering and hindering factors of reach, adoption, implementation and maintenance 15
References cited in Supplementary material 16
Additional references cited in main text 21

Table S1. Search syntax used for literature search in PubMed, PsycINFO and Web of Science

**PubMed**
(online[Title/Abstract] OR internet*[Title/Abstract] OR computer*[Title/Abstract] OR web*[Title/Abstract] OR e-mental health[Title/Abstract] OR e-health[Title/Abstract] OR "ehealth"[Title/Abstract] OR technol*[Title/Abstract] OR digital[Title/Abstract] OR mobile[Title/Abstract] OR smartphone[Title/Abstract] OR tablet[Title/Abstract] OR blended[Title/Abstract])<br>AND<br>(program*[Title/Abstract] OR intervention[Title/Abstract] OR application[Title/Abstract] OR app[Title/Abstract] OR self-help[Title/Abstract] OR prevent*[Title/Abstract] OR health promotion[Title/Abstract])<br>AND<br>(eating disorder[Title/Abstract] OR anorexia[Title/Abstract] OR bulimia[Title/Abstract] OR binge eating[Title/Abstract] OR EDNOS[Title/Abstract] OR OSFED[Title/Abstract] OR disordered eating[Title/Abstract]OR restrained eating[Title/Abstract] OR eating pathology[Title/Abstract] OR chronic diet*[Title/Abstract] OR body dissatisfaction[Title/Abstract] OR intuitive eating[Title/Abstract] OR weight regulation[Title/Abstract] OR body image[Title/Abstract] OR eating behavior[Title/Abstract] OR eating behaviour[Title/Abstract])
**PsycINFO**
((online OR internet OR computer* OR web* OR e-mental health OR e-health OR ehealth OR technol* OR digital OR mobile OR smartphone OR tablet OR blended)*<br>*AND*<br>*(program* OR intervention OR application OR app OR self-help OR prevent* OR health promotion)*<br>*AND*<br>*(eating disorder OR anorexia OR bulimia OR binge eating OR EDNOS OR OSFED OR disordered eating OR restrained eating OR eating pathology OR chronic diet* OR body dissatisfaction OR intuitive eating OR weight regulation OR body image OR eating behavior OR eating behaviour)).ti,ab.*
**Web of Science**
(TS=(online) OR TS=(internet*) OR TS=(computer*) OR TS=(web*) OR TS=("e-mental health") OR TS=("e-health") OR TS=("ehealth") OR TS=(technol*) OR TS=(digital) OR TS=(mobile) OR TS=(smartphone) OR TS=(tablet) OR TS=(blended))<br>AND<br>(TS=(program*) OR TS=(intervention) OR TS=(application) OR TS=(app) OR TS=(self-help) OR TS=(prevent*) OR TS=("health promotion"))<br>AND<br>(TS=("eating disorder") OR TS=(anorexia) OR TS=(bulimia) OR TS=("binge eating") OR TS=(EDNOS) OR TS=(OSFED) OR TS=("disordered eating") OR TS=("restrained eating") OR TS=("eating pathology") OR TS=("chronic diet*") OR TS=("body dissatisfaction") OR TS=("intuitive eating") OR TS=("weight regulation") OR TS=("body image") OR TS=("eating behavior") OR TS=("eating behaviour"))

Table S2. Adapted RE-AIM coding sheet used for the present systematic review

Item	Description
**General study and intervention characteristics**	
First author and publication year	
Country / Countries of intervention delivery	
Level of prevention	Universal, selected or indicated prevention[a]
Programme name	Name of the Internet-based intervention, if given
Theoretical background of intervention	E.g. CBT, psychoeducational
Main aim of intervention	E.g. reducing weight / shape concerns
Tailored programme (yes vs. no)	Tailoring means that the content of the programme is (partly) individualised to fit the user needs
Study arms	Number and type of study arms and description of comparison group(s)
Type of study	E.g. RCT, quasi-experimental study, uncontrolled pre-post study
**REACH**	
R1. Method to identify target population	Process by which target population was identified for participation in the intervention
R2. Inclusion / Exclusion criteria	Description of inclusion / exclusion criteria for individual participants
R3. Exclusion rate	% of participants excluded by exclusion criteria
R4. Sample size	Number of participants in study
R5. Participation rate / uptake rate	% of individuals who participated in intervention or study, whereby denominator is reported (e.g. number of approached individuals for uptake rate, or number of eligible individuals for participation rate)
R6. Characteristics of participants	% females, mean age, age range, other characteristics
R7. Characteristics of non-participants	% females, mean age, age range, other characteristics
R8. Representativeness of participants	Comparison of characteristics between participants and non-participants
R9. Reasons for declining participation	Description of reasons
R10. Recruitment strategies	Strategies to recruit individual participants
**EFFICACY / EFFECTIVENESS**	
E1. Measures and results for post-intervention assessment	Description of outcome measures
E2. Intention-to-treat analysis utilised	Analysing participants in the groups in which they were randomised regardless of adherence
E3. Imputation procedure	Description of imputation procedure used for Intention-to-treat analysis
E4. Quality of Life measure included	Description of use and results for quality of life
E5. Measure of satisfaction with / acceptability of programme[b]	Description of included measures regarding satisfaction and acceptability
E6. Effects at follow-up	At least one follow-up assessment (after post-intervention assessment) included
E7. Attrition	Description of type and % of attrition

**ADOPTION**	
Setting-based[c]	Is the intervention setting based? (for recruitment only, for intervention delivery only, for recruitment and intervention delivery, not setting-based)
A1. Type(s) of included settings[d]	Description of type of setting(s) (e.g. schools)
A2. Geographical characteristics of setting[d]	Description of where the intervention was delivered
A3. Inclusion and exclusion criteria for settings[d]	Description of eligibility criteria for settings
A4. Adoption rate[d]	% approached of eligible settings, % of settings participated of approached settings
A5. Characteristics of approached setting[d]	Description of main characteristics of settings approached
A6. Characteristics of non-approached settings[d]	Description of main characteristics of settings not approached
A7. Representativeness of participating settings[d]	Comparison of characteristics between approached and non-approached settings
A8. Reasons for declining of settings[d]	Description of reasons
Delivery agent/gatekeeper necessary to delivery intervention[e]	Yes, delivery agent necessary to deliver (parts of) the intervention; no, only gatekeeper necessary; no
A9. Method to identify delivery agent[f]	Description of process by which staff was identified for delivery of intervention
A10. Description of staff delivering intervention[f]	Characteristics of staff
A11. Level of expertise of delivery agent[f]	Description of expertise (e.g. training) of delivery agents
A12. Start-up costs	Description of costs for intervention set-up
**IMPLEMENTATION**	
I1. Format of intervention[g]	E.g. web-based only, blended intervention
I2. Frequency and intensity of intervention	Description of intervention duration and frequency
I3. Level / Type of staff support needed[h]	Description of tasks done by staff to deliver intervention
I4. Electronic devices used[i]	Electronic devices used to complete programme
I5. Extent to which intervention was delivered as intended	Description of individual adherence, compliance, attendance and/or staff adherence
I6. Consistency of intervention delivery	Description of consistency across staff, time, settings and subgroups of participants
I7. Costs of delivery	Description of costs (time, money)
I8. Incentives used[j]	Description of incentives for participation in study
I9. Data protection measures[k]	Description of data protection measures that were implemented
**MAINTENANCE**	
M1. Assessed outcomes ≥ 6 months	Follow-up periods ≥ 6 months post-intervention
M2. Drop-out rate to last follow-up[l]	% drop-out (if follow-up assessment ≥ 6 months)
M3. Current status of programme	Description of current status after end of study / funding period
M4. Adaptations made	Description of adaptations made to foster sustainability of programme

M5. Costs of maintenance	Description of costs (money, time) related to sustainability of programme

[a] We follow the terminology proposed by O'Connell et al.[1]: *Universal* preventive interventions are usually targeted to the general public or a whole population that has not been identified on the basis of individual eating disorder risk. *Selective* preventive interventions are targeted to individuals or a population subgroup whose risk of developing eating disorders is significantly higher than average. *Indicated* preventive interventions are targeted to high-risk individuals who are identified as having signs or symptoms foreshadowing an eating disorder but dot meet diagnostic levels at the current time. Studies that included individuals with elevated eating disorder-related measures (e.g. weight and shape concerns) were categorised as indicated prevention.
[b] This item was newly added as published guidelines for reporting Internet-based interventions recommend an extended evaluation of user acceptance and satisfaction.[2]
[c] This item was newly added as not all Internet-based interventions may be implemented in a specific setting. This item is not used for calculating overall reporting rates.
[d] coded only if the intervention was setting-based (either for recruitment, delivery or both); Explanation: As some online interventions are not meant to be implemented in a specific setting (e.g. online-only interventions) these setting-specific RE-AIM indicators are coded only if the intervention/recruitment is setting-based. Otherwise, the resulting (overall) reporting rates would be biased.
[e] This item was newly added as a delivery agent may not be needed for all Internet-based interventions. This item is not used for calculating overall reporting rates.
[f] coded only if a delivery agent/gatekeeper was needed; Explanation: As online interventions may be completely unguided, a delivery agent may not be needed, RE-AIM indicators referring to delivery agents/gatekeepers are coded only if one is needed. Otherwise, the resulting (overall) reporting rates would be biased.
[g] This item was newly added as the format of intervention (e.g. web-based only, blended) is an essential feature of Internet-based interventions which also may affect outcomes and attrition.[3]
[h] This item was newly added as published guidelines for reporting Internet-based interventions recommend an extended reporting of the level of needed staff support.[2]
[i] This item was newly added as compatibility with and use of mobile phones may affect adherence to Internet-based interventions. Thus, reporting of the electronic devices used to access Internet-based interventions should be reported.[4]
[j] This item was newly added as many researcher provide incentives for participants of Internet-based interventions as adherence[5] or outcomes[6] may be influenced. Thus, reporting the incentives given is highly recommended to be reported in studies evaluating Internet-based interventions.
[k] This item was newly added as published guidelines for reporting Internet-based interventions recommend an extended reporting of data protection measures.[2]
[l] coded only if M1 = yes

Table S3. Study and intervention characteristics of included studies and RE-AIM reporting rates of included studies

Study characteristics			Intervention characteristics				RE-AIM reporting rates (%)[a]					
Reference	Country	Study type & arms[b]	Intervention name	Level of prevention	Theoretical framework[c]	Tailoring	R	E	A	I	M	Total
						Number of indicators:	10	7	max. 12	9	max. 5	max. 43
Albertson et al. (2015)[7]	USA + Canada, UK, Australia, other (open trial)	RCT; IG, WLCG		Universal	Self-compassion mediation, mindfulness	No	50	43	0	44	25	42
Alleva et al. (2015)[8]	The Netherlands	RCT; IG, active CG	Expand Your Horizon	Indicated	Body functionality, objectification theory	No	70	71	11	56	0	46
Alleva et al. (2018a)[9]	UK (England)	RCT; IG, active CG	Expand Your Horizon	Universal	Body functionality, objectification theory	No	50	71	22	56	0	44
Alleva et al. (2018b)[10]	UK	RCT; IG, WLCG	Expand Your Horizon	Selected	Body functionality, objectification theory	No	60	86	0	56	0	55
Bauer et al. (2009)[11]	Germany	Pilot study; IG	ES[S]PRIT	Indicated	Psychoeducation	Yes[d]	80	0	22	44	25	38
Bedrosian et al. (2011)[12]	USA	Cross-sectional study; IG	-	Indicated, universal	[Not reported]	[Not reported]	40	0	11	0	0	13
Boucher et al. (2016)[13]	New Zealand	Pilot study; IG	Mind, Body, Food	Selected	Acceptance and commitment therapy, intuitive eating	No	80	100	11	78	0	59
Celio et al. (2000)[14]	USA	RCT; 1 IG Internet-based, 1 IG face-to-face, WLCG	Student Bodies	Universal	CBT exercises	No	50	71	33	78	40	55
Chithambo & Huey (2017)[15]	USA	RCT; 2 IGs Internet-based, CG	-	Selected	1 arm cognitive dissonance; 1 arm CBT	No	70	43	33	56	0	46
Cole et al. (2019)[16]	USA	Pilot pre-post study; 1 IG Internet-based, 1 IG face-to-face	My Body Knows When (MBKW)	Selected	Intuitive eating	No	40	29	17	33	0	26
Fitzsimmons-Craft et al. (2017)[17, e]	USA	RCT; IG, WLCG	Image and Mood (IaM)	Indicated	CBT, psychoeducation, interpersonal psychotherapy	No	50	14	11	44	40	33
Fitzsimmons-Craft et al. (2019)[18]	USA	Implementation study; 3 Internet-based IGs, 1 treatment referral group	StayingFit, Student Bodies - Classic, Student Bodies - Eating Disorders	Universal, selected, indicated	CBT	Yes[d]	70	29	67	67	25	57

Study characteristics			Intervention characteristics				RE-AIM reporting rates (%)[a]					
Reference	Country	Study type & arms[b]	Intervention name	Level of prevention	Theoretical framework[c]	Tailoring	R	E	A	I	M	Total
Franko et al. (2005)[19]	USA	RCT; IG, active CG	Food, Mood and Attitude (FMA)	Universal, selected	Dual pathway model of eating disorders (thin-ideal internalization), interpersonal theory, CBT	No	80	71	22	67	0	54
Franko & George (2008)[20]	USA	Pilot study; IG	Food, Mood and Attitude (FMA)	Selected	Dual pathway model of eating disorders (thin-ideal internalization), interpersonal theory, CBT	No	80	43	33	56	40	53
Franko et al. (2012)[21]	USA	RCT; IG, CG	Food, Mood and Attitude (FMA), MyStudentBody.com-Nutrition	Selected	[Not reported]	No	60	71	22	56	0	46
Geraghty et al. (2010)[22]	UK	RCT; 2 Internet-based IGs, 2 WLCGs	-	Universal	CBT, cognitive restructuring, positive psychology, gratitude	No	40	57	0	56	0	42
Gollings & Paxton (2006)[23]	Australia	RT, pilot study; 1 IG Internet-based, 1 IG face-to-face	Set Your Body Free	Indicated	CBT, motivational interviewing, psychoeducation	No	60	71	25	44	0	43
Jacobi et al. (2005)[24, f], Jacobi et al. (2007)[25, f]	Germany	RCT; IG, WLCG	StudentBodies	Universal	CBT, psychoeducation	No	70	57	22	67	25	51
Jacobi et al. (2012)[26, g] Völker et al. (2014)[27, g]	Germany	RCT; IG, WLCG	StudentBodies+	Indicated	CBT, psychoeducation	No	70	57	25	44	40	47
Järvelä-Reijonen et al. (2018)[28, h], Sairanen (2017)[29, h]	Finland	RCT; 1 IG Internet-based, 1 IG face-to-face, WLCG	Oiva	Selected	Acceptance and commitment therapy, mindfulness, intuitive eating	No	60	71	8	67	40	47
Jones et al. (2014)[30]	USA	Feasibility study; 2 Internet-based IGs, 1 blended (internet & face-to-face) IG, 1 clinical referral group	Healthy Body Image Program (HBI), including Staying Fit, StudentBodies	Universal, selected, indicated	CBT	Yes[d]	60	14	8	56	50	36
Kass et al. (2014)[31]	USA	RCT; 2 IGs	StudentBodies	Indicated	CBT, psychoeducation	No	80	57	22	67	25	54
Kattelmann et al. (2014)[32]	USA	RCT; IG, WLCG	Young Adults Eating and Active for Health (YEAH)	Universal	Transtheoretical Model of Behavior Change	Yes[i]	80	57	22	56	40	53

Study characteristics			Intervention characteristics				RE-AIM reporting rates (%)[a]					
Reference	Country	Study type & arms[b]	Intervention name	Level of prevention	Theoretical framework[c]	Tailoring	R	E	A	I	M	Total
Kindermann et al. (2017)[33]	Germany	cross-sectional study; IG	ProYouth	Indicated, universal	Psychoeducation	Yes[h]	50	0	8	44	0	24
Kollei et al. (2017)[34]	Germany	RCT; IG, WLCG	Mindtastic Body Dissatisfaction app (MT-BD)	Indicated	Approach and avoidance training	No	60	57	11	56	0	41
Lindenberg et al. (2011)[35]	Ireland	Acceptability study; IG	Appetite for Life	Universal, selected, indicated	CBT, psychoeducation	Yes[d]	90	29	22	67	0	49
Lipson et al. (2017)[36]	USA	2-phase pilot study; 3 IGs, clinical referral group	Healthy Body Study (HBS), Healthy Body Image (HBI)	Universal, selected, indicated	[Not reported]	Yes[d]	60	0	33	22	0	28
Low et al. (2006)[37]	USA	RCT; 3 Internet-based IGs, CG	StudentBodies	Universal	CBT, psychoeducation	No	50	71	33	78	40	55
Manwaring et al. (2008)[38, k]	USA	RCT; IG, WLCG	StudentBodies	Indicated	CBT, psychoeducation	No	50	43	22	44	40	40
Mason et al. (2018)[39]	USA	Single arm pre-post study	-	Selected	Operant conditioning, mindfulness	No	70	29	22	56	0	41
Mensorio et al. (2019)[40]	Spain	RCT; IG + TAU, WLCG + TAU	Living Better	Selected	CBT, psychoeducation	No	60	71	8	56	40	44
Minarik et al. (2013)[41]	Germany	Dissemination study; IG	ProYouth	Indicated, universal	Psychoeducation	Yes[j]	60	29	50	56	50	50
Moessner et al. (2016)[42]	Germany	Observational study; IG	ProYouth	Indicated, universal	Psychoeducation	Yes[j]	50	14	25	44	0	31
Ohlmer et al. (2013)[43]	Germany	Pilot study; IG	StudentBodies-AN	Indicated	CBT	No	80	86	8	56	60	53
Paxton et al. (2007)[44]	Australia	RCT; 1 Internet-based IG, 1 face-to-face IG, WLCG	Set Your Body Free	Indicated	CBT, psychoeducation	No	80	57	25	56	40	57
Pennesi & Wade (2018)[45]	Australia	RCT, pilot study; 2 Internet-based IGs, CG	-	Indicated	Imagery rescripting; cognitive dissonance (perceived pressure to be thin, internalization of the thin-ideal)	No	60	71	11	67	0	46

Study characteristics			Intervention characteristics				RE-AIM reporting rates (%)[a]					
Reference	Country	Study type & arms[b]	Intervention name	Level of prevention	Theoretical framework[c]	Tailoring	R	E	A	I	M	Total
Rodgers et al. (2018)[46]	USA	RCT; IG, CG	BodiMojo	Universal	Self-compassion and sociocultural theory, gratitude	No	30	43	33	67	0	38
Rohde et al. (2017)[47, l], Shaw et al. (2016)[48, l], Stice et al. (2017)[49, l]	USA	RCT; 1 Internet-based IG, 2 face-to-face IGs, active CG	eBody Project	Indicated	Cognitive dissonance	No	80	86	22	56	40	58
Saekow et al. (2015)[50]	USA	RCT; IG, WLCG	StudentBodies-ED	Indicated	CBT	No	90	86	33	67	25	62
Serdar et al. (2014)[51]	USA	RCT; 1 Internet-based IG, 1 face-to-face IG, CG	-	Universal	Cognitive dissonance	No	70	57	42	67	0	52
Stice et al. (2012)[52, m] Stice et al. (2014)[53, m]	USA	RCT; 1 Internet-based IG, 1 face-to-face IG, 2 active CGs	eBody Project	Indicated	Cognitive dissonance	No	90	86	22	56	80	65
Taylor et al. (2006)[54, k]	USA	RCT; IG, WLCG	StudentBodies	Indicated	CBT, psychoeducation	No	70	43	44	67	40	55
Taylor et al. (2016)[55, e]	USA	RCT; IG, WLCG	Image and Mood (IaM)	Indicated	CBT, psychoeducation, interpersonal psychotherapy	No	80	57	33	78	60	60
Toole & Craighead (2016)[56]	USA	RCT; IG, WLCG	-	Universal, selected	Self-compassion mediation, mindfulness	No	30	57	11	56	25	36
Völker et al. (2011)[57]	Germany	pilot study; IG	StudentBodies+	Indicated	CBT, psychoeducation	No	70	71	8	44	25	43
Walsh et al. (2017)[58]	USA	Quasi-experimental non-randomised feasibility study; IG, CG		Selected	Participatory research for development, non-diet weight management approach	Yes[j]	50	57	25	67	0	43
Weineland et al. (2012)[59]	Sweden	RCT; IG, TAU CG	-	Selected	Acceptance and commitment therapy	No	70	71	17	33	0	40
Wilksch et al. (2018)[60]	Australia, New Zealand	RCT; 2 Internet-based IGs, CG	Media Smart-Targeted (MS-T), StudentBodies (SB)	Universal	Cognitive dissonance (media internalization), CBT	No	70	86	0	67	40	66

Study characteristics			Intervention characteristics				RE-AIM reporting rates (%)[a]					
Reference	Country	Study type & arms[b]	Intervention name	Level of prevention	Theoretical framework[c]	Tailoring	R	E	A	I	M	Total
Winzelberg et al. (2000)[61]	USA	RCT; IG, WLCG	StudentBodies	Universal	CBT, psychoeducation	No	40	86	33	78	0	51
Yager & O'Dea (2010)[62]	Australia	Quasi-experimental non-randomised study; 1 blended (Internet-based and face-to-face components) IG, face-to-face IG, TAU CG	-	Selected	Theory of Planned Behavior, Social Learning Theory, Social Cognitive Theory, cognitive dissonance theory	No	40	43	8	33	40	30
Zabinski et al. (2001a)[63]	USA	RCT; IG, WLCG	StudentBodies	Indicated	CBT, psychoeducation	No	50	57	33	78	0	49
Zabinski et al. (2001b)[64]	USA	Pilot feasibility study; IG	-	Indicated	CBT	No	50	57	42	89	0	52
Zabinski et al. (2004)[65]	USA	RCT; IG, WLCG	-	Indicated	CBT, psychoeducation	No	80	86	42	89	0	64
Ziemer et al. (2019)[66]	USA	RCT; 2 Internet-based IGs, 1 CG	-	Universal	Self-compassion, body appreciation, expressive writing	No	50	57	33	44	0	41
					**Average reporting rates across studies (%)**		**63**	**54**	**24**	**57**	**22**	**46**

[a] R: reach; E: efficacy/effectiveness; A: adoption; I: implementation; M: maintenance.

[b] RCT: randomised controlled trial; RT: randomised trial; TAU: treatment as usual; IG: intervention group; WLCG: waitlist control group; CG: control group.

[c] CBT: cognitive behavioural therapy

[d] Segmented population, based on risk status

[e] The samples of these studies overlap. However, as they are not based on the exact same sample, they are treated as single studies.

[f] These articles refer to the same sample and study, so they are merged for the purpose of this review.

[g] These articles refer to the same sample and study, so they are merged for the purpose of this review.

[h] These articles refer to the same sample and study, so they are merged for the purpose of this review.

[i] Stage-based intervention nudges

[j] Self-chosen modules

[k] The samples of these studies overlap. However, as they are not based on the exact same sample, they are treated as single studies.

[l] These articles refer to the same sample and study, so they are merged for the purpose of this review.

[m] These articles refer to the same sample and study, so they are merged for the purpose of this review.

Table S4. Reporting status of RE-AIM indicators for each included study

Reference	Reach										Efficacy / Effectiveness							Adoption												Implementation									Maintenance				
	R1	R2	R3	R4	R5	R6	R7	R8	R9	R10	E1	E2	E3	E4	E5	E6	E7	A1	A2	A3	A4	A5	A6	A7	A8	A9	A10	A11	A12	I1	I2	I3	I4	I5	I6	I7	I8	I9	M1	M2	M3	M4	M5
Albertson et al. (2015)[7]	-	+	-	+	+	+	-	-	-	+	+	-	-	-	-	+	+	n.a.	n.a.	n.a.	n.a.	n.a.	n.a.	n.a.	n.a.	n.a.	n.a.	n.a.	-	+	+	-	-	+	-	-	+	-	-	n.a.	+	-	-
Alleva et al. (2015)[8]	+	+	+	+	+	+	-	-	-	+	+	+	+	-	-	+	+	+	-	-	-	-	-	-	-	n.a.	n.a.	n.a.	-	+	+	+	-	+	-	-	+	-	-	n.a.	-	-	-
Alleva et al. (2018a)[9]	-	+	-	+	+	+	-	-	-	+	+	+	+	-	-	+	+	+	+	-	-	-	-	-	-	n.a.	n.a.	n.a.	-	+	+	+	-	+	-	-	+	-	-	n.a.	-	-	-
Alleva et al. (2018b)[10]	-	+	-	+	+	+	-	+	-	+	+	+	+	+	-	+	+	n.a.	n.a.	n.a.	n.a.	n.a.	n.a.	n.a.	n.a.	n.a.	n.a.	n.a.	-	+	+	+	-	+	-	-	+	-	-	n.a.	-	-	-
Bauer et al. (2009)[11]	+	+	+	+	+	+	-	+	-	+	-	-	-	-	-	-	-	+	+	-	-	-	-	-	-	n.a.	n.a.	n.a.	-	+	+	+	-	+	-	-	-	-	-	n.a.	+	-	-
Bedrosian et al. (2011)[12]	+	-	-	+	-	+	-	-	-	+	-	-	-	-	-	-	-	+	-	-	-	-	-	-	-	n.a.	n.a.	n.a.	-	-	-	-	-	-	-	-	-	-	-	n.a.	-	-	-
Boucher et al. (2016)[13]	+	+	+	+	+	+	-	+	-	+	+	+	+	+	+	+	+	-	+	-	-	-	-	-	-	n.a.	n.a.	n.a.	-	+	+	+	+	+	-	-	+	+	-	+	-	-	-
Celio et al. (2000)[14]	-	+	-	+	-	+	-	+	-	+	+	+	+	-	-	+	+	+	+	-	-	+	-	-	-	n.a.	n.a.	n.a.	-	+	+	+	+	+	-	-	+	+	+	+	-	-	-
Chithambo & Huey (2017)[15]	+	+	+	+	+	+	-	-	-	+	+	-	+	-	-	-	+	+	+	-	-	+	-	-	-	n.a.	n.a.	n.a.	-	+	+	+	-	+	-	-	+	-	-	n.a.	-	-	-
Cole et al. (2019)[16]	-	+	-	+	-	+	-	-	-	+	+	-	-	-	-	-	+	+	+	-	-	-	-	-	-	-	-	-	-	+	+	+	-	-	-	-	-	-	-	n.a.	-	-	-
Fitzsimmons-Craft et al. (2017)[17]	+	+	-	+	-	+	-	-	-	+	-	-	-	-	-	-	+	+	-	-	-	-	-	-	-	n.a.	n.a.	n.a.	-	+	+	+	-	-	+	-	-	-	+	+	-	-	-
Fitzsimmons-Craft et al. (2019)[18]	+	+	-	+	+	+	-	+	-	+	+	-	-	-	-	-	+	+	+	+	+	+	-	-	+	-	+	+	-	+	+	+	+	+	+	-	-	-	-	n.a.	-	+	-
Franko et al. (2005)[19]	+	+	+	+	+	+	-	-	+	+	+	+	-	-	+	+	+	+	+	-	-	-	-	-	-	n.a.	n.a.	n.a.	-	+	+	+	+	-	-	-	+	+	-	n.a.	-	-	-
Franko & George (2008)[20]	+	+	+	+	+	+	-	-	+	+	+	-	-	-	-	+	+	+	+	-	-	+	-	-	-	n.a.	n.a.	n.a.	-	+	+	+	+	-	-	-	+	-	+	+	-	-	-
Franko et al. (2012)[21]	-	+	-	+	+	+	-	-	+	+	+	+	+	-	-	+	+	+	+	-	-	-	-	-	-	n.a.	n.a.	n.a.	-	+	+	+	+	-	-	-	+	-	-	n.a.	-	-	-
Geraghty et al. (2010)[22]	-	+	-	+	-	+	-	-	-	+	+	+	+	-	-	-	+	n.a.	n.a.	n.a.	n.a.	n.a.	n.a.	n.a.	n.a.	n.a.	n.a.	n.a.	-	+	+	+	-	+	-	-	+	-	-	n.a.	-	-	-

Reference	Reach										Efficacy / Effectiveness							Adoption												Implementation									Maintenance				
	R1	R2	R3	R4	R5	R6	R7	R8	R9	R10	E1	E2	E3	E4	E5	E6	E7	A1	A2	A3	A4	A5	A6	A7	A8	A9	A10	A11	A12	I1	I2	I3	I4	I5	I6	I7	I8	I9	M1	M2	M3	M4	M5
Gollings & Paxton (2006)[23]	+	+	+	+	-	+	-	-	-	+	+	+	+	-	-	+	+	+	+	-	-	-	-	-	-	-	+	-	-	+	+	+	-	-	-	-	-	+	-	n.a.	-	-	-
Jacobi et al. (2005)[24], Jacobi et al. (2007)[25]	+	+	+	+	+	+	-	-	-	+	+	-	-	-	+	+	+	+	+	-	-	-	-	-	-	n.a.	n.a.	n.a.	-	+	+	-	+	+	-	-	+	+	-	n.a.	+	-	-
Jacobi et al. (2012)[26] Völker et al. (2014)[27]	+	+	+	+	+	+	-	-	-	+	+	-	-	-	+	+	+	+	-	-	-	-	-	-	-	-	+	+	-	+	+	+	-	+	-	-	-	-	+	+	-	-	-
Järvelä-Reijonen et al. (2018)[28], Sairanen (2017)[29]	+	+	-	+	+	+	-	-	-	+	+	+	+	-	-	+	+	-	+	-	-	-	-	-	-	-	-	-	-	+	+	+	+	+	+	-	-	-	+	+	-	-	-
Jones et al. (2014)[30]	+	+	-	+	+	+	-	-	-	+	-	-	-	-	+	-	-	+	-	-	-	-	-	-	-	-	-	-	-	+	+	+	+	-	-	-	-	+	-	n.a.	+	+	-
Kass et al. (2014)[31]	+	+	+	+	+	+	-	-	+	+	+	+	+	-	-	-	+	+	+	-	-	-	-	-	-	n.a.	n.a.	n.a.	-	+	+	+	-	+	-	+	-	+	-	n.a.	+	-	-
Kattelmann et al. (2014)[32]	+	+	+	+	+	+	-	+	-	+	+	-	-	-	+	+	+	+	+	-	-	-	-	-	-	n.a.	n.a.	n.a.	-	+	+	-	-	+	-	-	+	+	+	+	-	-	-
Kindermann et al. (2017)[33]	+	+	-	+	-	+	-	-	-	+	-	-	-	-	-	-	-	+	-	-	-	-	-	-	-	-	-	-	-	+	+	+	-	+	-	-	-	-	-	n.a.	-	-	-
Kollei et al. (2017)[34]	+	+	-	+	-	+	-	+	-	+	+	+	+	-	-	+	-	+	-	-	-	-	-	-	-	n.a.	n.a.	n.a.	-	+	+	+	+	-	-	-	+	-	-	n.a.	-	-	-
Lindenberg et al. (2011)[35]	+	+	+	+	+	+	+	+	-	+	-	-	-	-	+	-	+	+	+	-	-	-	-	-	-	n.a.	n.a.	n.a.	-	+	+	+	-	+	+	-	-	+	-	n.a.	-	-	-
Lipson et al. (2017)[36]	+	+	-	+	+	+	-	-	-	+	-	-	-	-	-	-	-	+	+	-	-	+	-	-	-	n.a.	n.a.	n.a.	-	+	-	-	-	-	-	-	+	-	-	n.a.	-	-	-
Low et al. (2006)[37]	-	+	-	+	+	+	-	-	-	+	+	+	+	-	-	+	+	+	+	-	-	+	-	-	-	n.a.	n.a.	n.a.	-	+	+	+	+	+	-	-	+	+	+	+	-	-	-
Manwaring et al. (2008)[38]	+	+	-	+	-	+	-	-	-	+	+	-	-	-	-	+	+	+	+	-	-	-	-	-	-	n.a.	n.a.	n.a.	-	+	+	+	-	+	-	-	-	-	+	+	-	-	-
Mason et al. (2018)[39]	+	+	+	+	+	+	-	-	-	+	+	-	-	-	-	-	+	+	+	-	-	-	-	-	-	n.a.	n.a.	n.a.	-	+	+	-	+	+	+	-	-	-	-	n.a.	-	-	-
Mensorio et al. (2019)[40]	-	+	+	+	+	+	-	-	-	+	+	+	-	+	-	+	+	+	-	-	-	-	-	-	-	-	-	-	-	+	+	+	+	+	-	-	-	-	+	+	-	-	-

Reference	Reach										Efficacy / Effectiveness							Adoption												Implementation									Maintenance				
	R1	R2	R3	R4	R5	R6	R7	R8	R9	R10	E1	E2	E3	E4	E5	E6	E7	A1	A2	A3	A4	A5	A6	A7	A8	A9	A10	A11	A12	I1	I2	I3	I4	I5	I6	I7	I8	I9	M1	M2	M3	M4	M5
Minarik et al. (2013)[41]	+	+	-	+	+	+	-	-	-	+	-	-	-	-	+	-	+	+	+	-	+	-	-	-	-	+	+	+	-	+	+	+	-	+	-	+	-	-	-	n.a.	+	-	+
Moessner et al. (2016)[42]	+	+	-	+	-	+	-	-	-	+	+	-	-	-	-	-	-	+	-	-	-	-	-	-	-	-	+	+	-	+	+	+	-	+	-	-	-	-	-	n.a.	-	-	-
Ohlmer et al. (2013)[43]	+	+	+	+	+	+	-	+	-	+	+	+	+	-	+	+	+	+	-	-	-	-	-	-	-	-	-	-	-	+	+	+	-	+	-	-	+	-	+	+	+	-	-
Paxton et al. (2007)[44]	+	+	+	+	-	+	-	+	+	+	+	+	-	-	-	+	+	n.a.	n.a.	n.a.	n.a.	n.a.	n.a.	n.a.	n.a.	-	+	-	-	+	+	+	-	+	-	-	-	+	+	+	-	-	-
Pennesi & Wade (2018)[45]	+	+	+	+	+	+	-	-	-	-	+	+	+	+	-	-	+	+	-	-	-	-	-	-	-	n.a.	n.a.	n.a.	-	+	+	+	+	+	-	-	+	-	-	n.a.	-	-	-
Rodgers et al. (2018)[46]	-	-	-	+	-	+	-	-	-	+	+	-	-	-	-	+	+	+	+	-	-	+	-	-	-	n.a.	n.a.	n.a.	-	+	+	+	+	-	-	-	+	+	-	n.a.	-	-	-
Rohde et al. (2017)[47], Shaw et al. (2016)[48], Stice et al. (2017)[49]	+	+	+	+	+	+	-	-	+	+	+	+	+	-	+	+	+	+	+	-	-	-	-	-	-	n.a.	n.a.	n.a.	-	+	+	+	-	+	-	-	+	-	+	+	-	-	-
Saekow et al. (2015)[50]	+	+	+	+	+	+	-	+	+	+	+	+	+	+	+	-	+	+	-	-	-	+	-	-	-	-	+	+	-	+	+	+	+	+	-	-	-	+	-	n.a.	+	-	-
Serdar et al. (2014)[51]	+	+	+	+	-	+	-	+	-	+	+	+	+	-	-	-	+	+	+	-	-	+	-	-	-	-	+	+	-	+	+	+	-	+	-	-	+	+	-	n.a.	-	-	-
Stice et al. (2012)[52] Stice et al. (2014)[53]	+	+	+	+	+	+	-	+	+	+	+	+	+	-	+	+	+	+	+	-	-	-	-	-	-	n.a.	n.a.	n.a.	-	+	+	+	-	+	-	-	+	-	+	+	+	+	-
Taylor et al. (2006)[54]	+	+	+	+	+	+	-	-	-	+	+	-	-	-	-	+	+	+	+	-	-	+	-	+	-	n.a.	n.a.	n.a.	-	+	+	+	+	+	-	+	-	-	+	+	-	-	-
Taylor et al. (2016)[55]	+	+	+	+	+	+	-	+	-	+	+	+	-	-	-	+	+	+	+	-	-	-	-	-	-	-	+	+	-	+	+	+	-	+	+	+	+	-	+	+	-	-	+
Toole & Craighead (2016)[56]	-	+	-	+	-	+	-	-	-	-	+	+	-	-	+	-	+	+	-	-	-	-	-	-	-	n.a.	n.a.	n.a.	-	+	+	+	-	+	-	-	+	-	-	n.a.	+	-	-
Völker et al. (2011)[57]	+	+	+	+	+	+	-	-	-	+	+	+	+	-	+	-	+	+	-	-	-	-	-	-	-	-	-	-	-	+	+	+	-	+	-	-	-	-	-	n.a.	+	-	-
Walsh et al. (2017)[58]	-	+	-	+	-	+	-	+	-	+	+	-	-	-	+	+	+	+	+	-	-	-	-	-	-	-	+	-	-	+	+	+	+	+	-	-	-	+	-	n.a.	-	-	-

Reference	Reach										Efficacy / Effectiveness							Adoption												Implementation									Maintenance				
	R1	R2	R3	R4	R5	R6	R7	R8	R9	R10	E1	E2	E3	E4	E5	E6	E7	A1	A2	A3	A4	A5	A6	A7	A8	A9	A10	A11	A12	I1	I2	I3	I4	I5	I6	I7	I8	I9	M1	M2	M3	M4	M5
Weineland et al. (2012)[59]	+	+	+	+	+	+	-	-	-	+	+	+	+	+	-	-	+	+	+	-	-	-	-	-	-	-	-	-	-	+	+	+	-	-	-	-	-	-	-	n.a.	-	-	-
Wilksch et al. (2018)[60]	+	+	+	+	+	+	-	-	-	+	+	+	+	+	-	+	+	n.a.	n.a.	n.a.	n.a.	n.a.	n.a.	n.a.	n.a.	n.a.	n.a.	n.a.	-	+	+	+	-	+	-	-	+	+	+	+	-	-	-
Winzelberg et al. (2000)[61]	-	+	-	+	-	+	-	-	-	+	+	+	+	-	+	+	+	+	+	-	-	+	-	-	-	n.a.	n.a.	n.a.	-	+	+	+	+	+	-	-	+	+	-	n.a.	-	-	-
Yager & O'Dea (2010)[62]	-	+	-	+	+	+	-	-	-	+	+	-	-	-	-	+	+	+	-	-	-	-	-	-	-	-	-	-	-	+	+	+	-	-	-	-	-	-	+	+	-	-	-
Zabinski et al. (2001a)[63]	+	+	-	+	-	+	-	-	-	+	+	-	-	-	+	+	+	+	+	-	-	+	-	-	-	n.a.	n.a.	n.a.	-	+	+	+	+	+	-	-	+	+	-	n.a.	-	-	-
Zabinski et al. (2001b)[64]	+	+	-	+	-	+	-	-	-	+	+	-	-	-	+	+	+	+	+	-	-	+	-	-	-	-	+	+	-	+	+	+	+	+	+	-	+	+	-	n.a.	-	-	-
Zabinski et al. (2004)[65]	+	+	+	+	+	+	-	-	+	+	+	+	+	-	+	+	+	+	+	-	-	+	-	-	-	-	+	+	-	+	+	+	+	+	+	-	+	+	-	n.a.	-	-	-
Ziemer et al. (2019)[66]	-	+	-	+	-	+	-	+	-	+	+	-	+	+	-	-	+	+	+	-	-	+	-	-	-	n.a.	n.a.	n.a.	-	+	+	+	-	-	-	-	+	-	-	n.a.	-	-	-

+ reported, - not reported, n.a. not applicable; for numbering of indicators see Table S2

Table S5. Overview of fostering and hindering factors of reach, adoption, implementation and maintenance

Fostering factors	Hindering factors
**REACH**	
• Anonymous, low-threshold access of Internet-based interventions[12,20,21,30,34,35,44]<br>• flexible use independent of time and geographical location[12,14,62,63]<br>• No costs for participants[35,56]<br>• Embedding recruitment in an educational setting (university campus[18], or vocational training[58])	• Recruitment strategies used in study possibly unsuitable to reach a diverse population or the population most in need for the intervention[10,23,47]<br>• Computer/technology literacy might have posed a barrier for some participants[23,54]<br>• Interventions available for one operating system only[18]
**ADOPTION**	
• Internet-based format of the intervention (vs. having to identify university clinicians to deliver the intervention)[52]<br>• College infrastructure[49]<br>• Making ED screenings mandatory in a college setting in future implementation efforts[18]	• Potential costs for screening and treatment at campuses[18]<br>• Not meeting participant's preferences of online vs. offline intervention[48]
**IMPLEMENTATION**	
• Low implementation costs[8,10,16,19,55]<br>• Feasible[8,10,16,41,63] and time-saving[36,66] delivery of intervention<br>• Flexible, self-directed use[14,19,35,41,48,50,66]<br>• Providing access to Internet/computers[37]<br>• Fostering engagement of participants: discussion groups,[14,23,31,62] anonymous participation,[41,44] and electronic reminders,[55,60] self-selected samples,[27,65] and high motivation of participants[54]<br>• Implementation at a vocational training site[58]<br>• Combining screening and intervention[30,36]	• technical problems during the study,[9,13,44,50,64,65] usability issues,[13,31,36,51,60] and restricted access to computers[58]<br>• Hindering engagement: computer competency of participants,[40,44,54,64,65] delay between screening and start of intervention,[36,50] lack of personal interaction,[44,48,60,61] privacy concerns,[37,64] time constraints of participants,[13,14,50,56] motivational problems,[13,20,39,51,57] not meeting participants needs and interests,[13,24,40,50] health problems,[13,40] family issues,[40] an all-female staff in a mixed-gender intervention,[41] and lower commitment due to anonymity[35]<br>• Participants' withdrawals and staffing challenges[16]
**MAINTENANCE**	
• High potential to feasibly disseminate an existing intervention to further settings, e.g. health services or educational courses[10,12,21,26,30,34,47,62-64]<br>• Sponsoring,[18] high stakeholder involvement,[31] and promotion of programmes[41]<br>• Specific intervention features: automated components and procedures,[18,60] coaches dashboards,[50] and manuals[23,31]	• Limited funding and staff resources[18,31]

References cited in Supplementary material

1. O'Connell ME, Boat T, Warner KE. Defining the scope of prevention [Internet]. In: *Preventing Mental, Emotional, and Behavioral Disorders Among Young People: Progress and Possibilities.* Washington (DC): National Academies Press (US); 2009.

2. Proudfoot J, Klein B, Barak A, et al. Establishing guidelines for executing and reporting internet intervention research. *Cognitive Behaviour Therapy.* 2011;40(2):82-97.

3. Erbe D, Eichert HC, Riper H, Ebert DD. Blending Face-to-Face and Internet-Based Interventions for the Treatment of Mental Disorders in Adults: Systematic Review. *J Med Internet Res.* 2017;19(9):e306.

4. Juarascio AS, Manasse SM, Goldstein SP, Forman EM, Butryn ML. Review of smartphone applications for the treatment of eating disorders. *European Eating Disorders Review.* 2015;23(1):1-11.

5. Fridrici M, Lohaus A, Glass C. Effects of incentives in web-based prevention for adolescents: Results of an exploratory field study. *Psychology and Health.* 2009;24(6):663-675.

6. Davies EB, Morriss R, Glazebrook C. Computer-delivered and web-based interventions to improve depression, anxiety, and psychological well-being of university students: a systematic review and meta-analysis. *J Med Internet Res.* 2014;16(5):e130.

7. Albertson ER, Neff KD, Dill-Shackleford KE. Self-Compassion and Body Dissatisfaction in Women: A Randomized Controlled Trial of a Brief Meditation Intervention. *Mindfulness.* 2015;6(3):444-454.

8. Alleva JM, Martijn C, Van Breukelen GJ, Jansen A, Karos K. Expand Your Horizon: A programme that improves body image and reduces self-objectification by training women to focus on body functionality. *Body Image.* 2015;15:81-89.

9. Alleva JM, Diedrichs PC, Halliwell E, et al. A randomised-controlled trial investigating potential underlying mechanisms of a functionality-based approach to improving women's body image. *Body Image.* 2018;25:85-96.

10. Alleva JM, Diedrichs PC, Halliwell E, et al. More than my RA: A randomized trial investigating body image improvement among women with rheumatoid arthritis using a functionality-focused intervention program. *J Consult Clin Psychol.* 2018;86(8):666-676.

11. Bauer S, Moessner M, Wolf M, Haug S, Kordy H. ES[S]PRIT - an Internet-based programme for the prevention and early intervention of eating disorders in college students. *Br J Guid Counc.* 2009;37(3):327-336.

12. Bedrosian RC, Striegel-Moore RH, Wang C. Demographic and clinical characteristics of individuals utilizing an internet-based digital coaching program for recovering from binge eating. *Int J Eat Disord.* 2011;44(7):639-646.

13. Boucher S, Edwards O, Gray A, et al. Teaching Intuitive Eating and Acceptance and Commitment

Therapy Skills Via a Web-Based Intervention: A Pilot Single-Arm Intervention Study. *JMIR Res Protoc.* 2016;5(4):e180.

14. Celio AA, Winzelberg AJ, Wilfley DE, et al. Reducing Risk Factors for Eating Disorders: Comparison of an Internetand a Classroom-Delivered Psychoeducational Program. *J Consult Clin Psychol.* 2000;68(4):650-657.

15. Chithambo TP, Huey SJ, Jr. Internet-delivered eating disorder prevention: A randomized controlled trial of dissonance-based and cognitive-behavioral interventions. *Int J Eat Disord.* 2017;50(10):1142-1151.

16. Cole RE, Meyer SA, Newman TJ, et al. The My Body Knows When Program Increased Intuitive Eating Characteristics in a Military Population. *Mil Med.* 2019;184(7-8):e200-e206.

17. Fitzsimmons-Craft EE, Eichen DM, Kass AE, et al. Reciprocal longitudinal relations between weight/shape concern and comorbid pathology among women at very high risk for eating disorder onset. *Eat Weight Disord.* 2017.

18. Fitzsimmons-Craft EE, Firebaugh ML, Graham AK, et al. State-wide university implementation of an online platform for eating disorders screening and intervention. *Psychol Serv.* 2019;16(2):239-249.

19. Franko DL, Mintz LB, Villapiano M, et al. Food, Mood, and Attitude: Reducing Risk for Eating Disorders in College Women. *Health Psychol.* 2005;24(6):567-578.

20. Franko DL, George JB. A pilot intervention to reduce eating disorder risk in Latina women. *Eur Eat Disord Rev.* 2008;16(6):436-441.

21. Franko DL, Jenkins A, Rodgers RF. Toward Reducing Risk for Eating Disorders and Obesity in Latina College Women. *J Couns Dev.* 2012;90(3):298-307.

22. Geraghty AW, Wood AM, Hyland ME. Attrition from self-directed interventions: investigating the relationship between psychological predictors, intervention content and dropout from a body dissatisfaction intervention. *Soc Sci Med.* 2010;71(1):30-37.

23. Gollings EK, Paxton SJ. Comparison of internet and face-to-face delivery of a group body image and disordered eating intervention for women: a pilot study. *Eat Disord.* 2006;14(1):1-15.

24. Jacobi C, Morris L, Beckers C, et al. Reduktion von Risikofaktoren für gestörtes Essverhalten: Adaptation und erste Ergebnisse eines Internet-gestützten Präventionsprogramms. *Z Gesundheitspsychol.* 2005;13(2):92-101.

25. Jacobi C, Morris L, Beckers C, et al. Maintenance of internet-based prevention: a randomized controlled trial. *Int J Eat Disord.* 2007;40(2):114-119.

26. Jacobi C, Völker U, Trockel MT, Taylor CB. Effects of an Internet-based intervention for subthreshold eating disorders: a randomized controlled trial. *Behav Res Ther.* 2012;50(2):93-99.

27. Völker U, Jacobi C, Trockel MT, Taylor CB. Moderators and mediators of outcome in Internet-based indicated prevention for eating disorders. *Behav Res Ther.* 2014;63:114-121.

28. Järvelä-Reijonen E, Karhunen L, Sairanen E, et al. The effects of acceptance and commitment therapy on eating behavior and diet delivered through face-to-face contact and a mobile app: a randomized controlled trial. *Int J Behav Nutr Phys Act.* 2018;15(1):22.

29. Sairanen E, Tolvanen A, Karhunen L, et al. Psychological flexibility mediates change in intuitive eating regulation in acceptance and commitment therapy interventions. *Public Health Nutr.* 2017;20(9):1681-1691.

30. Jones M, Kass AE, Trockel M, Glass AI, Wilfley DE, Taylor CB. A population-wide screening and tailored intervention platform for eating disorders on college campuses: the healthy body image program. *J Am Coll Health.* 2014;62(5):351-356.

31. Kass AE, Trockel M, Safer DL, et al. Internet-based preventive intervention for reducing eating disorder risk: A randomized controlled trial comparing guided with unguided self-help. *Behav Res Ther.* 2014;63:90-98.

32. Kattelmann KK, Bredbenner CB, White AA, et al. The effects of Young Adults Eating and Active for Health (YEAH): a theory-based Web-delivered intervention. *J Nutr Educ Behav.* 2014;46(6):S27-41.

33. Kindermann S, Moessner M, Ozer F, Bauer S. Associations between eating disorder related symptoms and participants' utilization of an individualized Internet-based prevention and early intervention program. *Int J Eat Disord.* 2017;50(10):1215-1221.

34. Kollei I, Lukas CA, Loeber S, Berking M. An app-based blended intervention to reduce body dissatisfaction: A randomized controlled pilot study. *J Consult Clin Psychol.* 2017;85(11):1104-1108.

35. Lindenberg K, Moessner M, Harney J, McLaughlin O, Bauer S. E-Health for Individualized Prevention of Eating Disorders. *Clin Pract Epidemiol Ment Health.* 2011;7:74-83.

36. Lipson SK, Jones JM, Taylor CB, et al. Understanding and promoting treatment-seeking for eating disorders and body image concerns on college campuses through online screening, prevention and intervention. *Eat Behav.* 2017;25:68-73.

37. Low KG, Charanasomboon S, Lesser J, et al. Effectiveness of a computer-based interactive eating disorders prevention program at long-term follow-up. *Eat Disord.* 2006;14(1):17-30.

38. Manwaring JL, Bryson SW, Goldschmidt AB, et al. Do adherence variables predict outcome in an online program for the prevention of eating disorders? *J Consult Clin Psychol.* 2008;76(2):341-346.

39. Mason AE, Jhaveri K, Cohn M, Brewer JA. Testing a mobile mindful eating intervention targeting craving-related eating: feasibility and proof of concept. *J Behav Med.* 2018;41(2):160-173.

40. Mensorio MS, Cebolla-Marti A, Rodilla E, et al. Analysis of the efficacy of an internet-based self-administered intervention ("Living Better") to promote healthy habits in a population with obesity and hypertension: An exploratory randomized controlled trial. *Int J Med Inform.* 2019;124:13-23.

41. Minarik C, Moessner M, Ozer F, Bauer S. [Implementation and dissemination of an internet-based program for prevention and early intervention in eating disorders]. *Psychiatr Prax.* 2013;40(6):332-338.

42. Moessner M, Minarik C, Ozer F, Bauer S. Can an internet-based program for the prevention and early intervention in eating disorders facilitate access to conventional professional healthcare? *J Ment Health.* 2016;25(5):441-447.

43. Ohlmer R, Jacobi C, Taylor CB. Preventing symptom progression in women at risk for AN: results of a pilot study. *Eur Eat Disord Rev.* 2013;21(4):323-329.

44. Paxton SJ, McLean SA, Gollings EK, Faulkner C, Wertheim EH. Comparison of face-to-face and internet interventions for body image and eating problems in adult women: an RCT. *Int J Eat Disord.* 2007;40(8):692-704.

45. Pennesi JL, Wade TD. Imagery rescripting and cognitive dissonance: A randomized controlled trial of two brief online interventions for women at risk of developing an eating disorder. *Int J Eat Disord.* 2018;51(5):439-448.

46. Rodgers RF, Donovan E, Cousineau T, et al. BodiMojo: Efficacy of a Mobile-Based Intervention in Improving Body Image and Self-Compassion among Adolescents. *J Youth Adolesc.* 2018;47(7):1363-1372.

47. Rohde P, Stice E, Shaw H, Gau JM, Ohls OC. Age effects in eating disorder baseline risk factors and prevention intervention effects. *Int J Eat Disord.* 2017;50(11):1273-1280.

48. Shaw H, Rohde P, Stice E. Participant feedback from peer-led, clinician-led, and internet-delivered eating disorder prevention interventions. *Int J Eat Disord.* 2016;49(12):1087-1092.

49. Stice E, Rohde P, Shaw H, Gau JM. Clinician-led, peer-led, and internet-delivered dissonance-based eating disorder prevention programs: Acute effectiveness of these delivery modalities. *J Consult Clin Psychol.* 2017;85(9):883-895.

50. Saekow J, Jones M, Gibbs E, et al. StudentBodies-eating disorders: A randomized controlled trial of a coached online intervention for subclinical eating disorders. *Internet Interv.* 2015;2(4):419-428.

51. Serdar K, Kelly NR, Palmberg AA, et al. Comparing online and face-to-face dissonance-based eating disorder prevention. *Eat Disord.* 2014;22(3):244-260.

52. Stice E, Rohde P, Durant S, Shaw H. A preliminary trial of a prototype Internet dissonance-based eating disorder prevention program for young women with body image concerns. *J Consult Clin Psychol.* 2012;80(5):907-916.

53. Stice E, Durant S, Rohde P, Shaw H. Effects of a prototype Internet dissonance-based eating disorder prevention program at 1- and 2-year follow-up. *Health Psychol.* 2014;33(12):1558-1567.

54. Taylor CB, Bryson S, Luce KH, et al. Prevention of Eating Disorders in At-Risk College-Age Women. *Arch Gen Psychiatry.* 2006;63(8):881-888.

55. Taylor CB, Kass AE, Trockel M, et al. Reducing eating disorder onset in a very high risk sample with significant comorbid depression: A randomized controlled trial. *J Consult Clin Psychol.* 2016;84(5):402-414.

56. Toole AM, Craighead LW. Brief self-compassion meditation training for body image distress in young adult women. *Body Image.* 2016;19:104-112.

57. Völker U, Jacobi C, Taylor CB. Adaptation and evaluation of an Internet-based prevention program for eating disorders in a sample of women with subclinical eating disorder symptoms: A pilot study. *Eat Weight Disord.* 2011;16(4):270-273.

58. Walsh J, Kattelmann K, White A. Stage-based healthy lifestyles program for non-college young adults. *Health Educ.* 2017;117(2):148-161.

59. Weineland S, Arvidsson D, Kakoulidis TP, Dahl J. Acceptance and commitment therapy for bariatric surgery patients, a pilot RCT. *Obes Res Clin Pract.* 2012;6(1):e1-e90.

60. Wilksch SM, O'Shea A, Taylor CB, Wilfley D, Jacobi C, Wade TD. Online prevention of disordered eating in at-risk young-adult women: a two-country pragmatic randomized controlled trial. *Psychol Med.* 2018;48(12):2034-2044.

61. Winzelberg AJ, Eppstein D, Eldredge KL, et al. Effectiveness of an Internet-Based Program for Reducing Risk Factors for Eating Disorders. *J Consult Clin Psychol.* 2000;68(2):346-350.

62. Yager Z, O'Dea J. A controlled intervention to promote a healthy body image, reduce eating disorder risk and prevent excessive exercise among trainee health education and physical education teachers. *Health Educ Res.* 2010;25(5):841-852.

63. Zabinski MF, Pung MA, Wilfley DE, et al. Reducing Risk Factors for Eating Disorders: Targeting At-Risk Women with a Computerized Psychoeducational Program. *Int J Eat Disord.* 2001;29(4):401-408.

64. Zabinski MF, Wilfley DE, Pung MA, Winzelberg AJ, Eldredge K, Taylor CB. An Interactive Internet-Based Intervention for Women at Risk of Eating Disorders: A Pilot Study. *Int J Eat Disord.* 2001;30(2):129-137.

65. Zabinski MF, Wilfley DE, Calfas KJ, Winzelberg AJ, Taylor CB. An interactive psychoeducational intervention for women at risk of developing an eating disorder. *J Consult Clin Psychol.* 2004;72(5):914-919.

66. Ziemer KS, Lamphere BR, Raque-Bogdan TL, Schmidt CK. A Randomized Controlled Study of Writing Interventions on College Women's Positive Body Image. *Mindfulness.* 2019;10(1):66-77.

Additional references cited in main text

41. Boucher S, Edwards O, Gray A, et al. Teaching Intuitive Eating and Acceptance and Commitment Therapy Skills Via a Web-Based Intervention: A Pilot Single-Arm Intervention Study. *JMIR Res Protoc.* 2016;5(4):e180.

42. Sairanen E, Tolvanen A, Karhunen L, et al. Psychological flexibility mediates change in intuitive eating regulation in acceptance and commitment therapy interventions. *Public Health Nutr.* 2017;20(9):1681-1691.

43. Kattelmann KK, Bredbenner CB, White AA, et al. The effects of Young Adults Eating and Active for Health (YEAH): a theory-based Web-delivered intervention. *J Nutr Educ Behav.* 2014;46(6):S27-41.

44. Ohlmer R, Jacobi C, Taylor CB. Preventing symptom progression in women at risk for AN: results of a pilot study. *Eur Eat Disord Rev.* 2013;21(4):323-329.

45. Jones M, Kass AE, Trockel M, Glass AI, Wilfley DE, Taylor CB. A population-wide screening and tailored intervention platform for eating disorders on college campuses: the healthy body image program. *J Am Coll Health.* 2014;62(5):351-356.

46. Zabinski MF, Pung MA, Wilfley DE, et al. Reducing Risk Factors for Eating Disorders: Targeting At-Risk Women with a Computerized Psychoeducational Program. *Int J Eat Disord.* 2001;29(4):401-408.

47. Jacobi C, Morris L, Beckers C, et al. Maintenance of internet-based prevention: a randomized controlled trial. *Int J Eat Disord.* 2007;40(2):114-119.

48. Wilksch SM, O'Shea A, Taylor CB, Wilfley D, Jacobi C, Wade TD. Online prevention of disordered eating in at-risk young-adult women: a two-country pragmatic randomized controlled trial. *Psychol Med.* 2018;48(12):2034-2044.

49. Toole AM, Craighead LW. Brief self-compassion meditation training for body image distress in young adult women. *Body Image.* 2016;19:104-112.

50. Walsh J, Kattelmann K, White A. Stage-based healthy lifestyles program for non-college young adults. *Health Educ.* 2017;117(2):148-161.

51. Gollings EK, Paxton SJ. Comparison of internet and face-to-face delivery of a group body image and disordered eating intervention for women: a pilot study. *Eat Disord.* 2006;14(1):1-15.

52. Rohde P, Stice E, Shaw H, Gau JM, Ohls OC. Age effects in eating disorder baseline risk factors and prevention intervention effects. *Int J Eat Disord.* 2017;50(11):1273-1280.

53. Taylor CB, Bryson S, Luce KH, et al. Prevention of Eating Disorders in At-Risk College-Age Women. *Arch Gen Psychiatry.* 2006;63(8):881-888.

54. Stice E, Rohde P, Durant S, Shaw H. A preliminary trial of a prototype Internet dissonance-based eating disorder prevention program for young women with body image concerns. *J Consult Clin Psychol.* 2012;80(5):907-916.

55. Stice E, Rohde P, Shaw H, Gau JM. Clinician-led, peer-led, and internet-delivered dissonance-based eating disorder prevention programs: Acute effectiveness of these delivery modalities. *J Consult Clin Psychol.* 2017;85(9):883-895.

56. Shaw H, Rohde P, Stice E. Participant feedback from peer-led, clinician-led, and internet-delivered eating disorder prevention interventions. *Int J Eat Disord.* 2016;49(12):1087-1092.

57. Franko DL, Mintz LB, Villapiano M, et al. Food, Mood, and Attitude: Reducing Risk for Eating Disorders in College Women. *Health Psychol.* 2005;24(6):567-578.

58. Albertson ER, Neff KD, Dill-Shackleford KE. Self-Compassion and Body Dissatisfaction in Women: A Randomized Controlled Trial of a Brief Meditation Intervention. *Mindfulness.* 2015;6(3):444-454.

59. Alleva JM, Martijn C, Van Breukelen GJ, Jansen A, Karos K. Expand Your Horizon: A programme that improves body image and reduces self-objectification by training women to focus on body functionality. *Body Image.* 2015;15:81-89.

60. Alleva JM, Diedrichs PC, Halliwell E, et al. A randomised-controlled trial investigating potential underlying mechanisms of a functionality-based approach to improving women's body image. *Body Image.* 2018;25:85-96.

61. Pennesi JL, Wade TD. Imagery rescripting and cognitive dissonance: A randomized controlled trial of two brief online interventions for women at risk of developing an eating disorder. *Int J Eat Disord.* 2018;51(5):439-448.

62. Ziemer KS, Lamphere BR, Raque-Bogdan TL, Schmidt CK. A Randomized Controlled Study of Writing Interventions on College Women's Positive Body Image. *Mindfulness.* 2019;10(1):66-77.

63. Kass AE, Trockel M, Safer DL, et al. Internet-based preventive intervention for reducing eating disorder risk: A randomized controlled trial comparing guided with unguided self-help. *Behav Res Ther.* 2014;63:90-98.

64. Cole RE, Meyer SA, Newman TJ, et al. The My Body Knows When Program Increased Intuitive Eating Characteristics in a Military Population. *Mil Med.* 2019;184(7-8):e200-e206.

65. Lipson SK, Jones JM, Taylor CB, et al. Understanding and promoting treatment-seeking for eating disorders and body image concerns on college campuses through online screening, prevention and intervention. *Eat Behav.* 2017;25:68-73.

66. Low KG, Charanasomboon S, Lesser J, et al. Effectiveness of a computer-based interactive eating disorders prevention program at long-term follow-up. *Eat Disord.* 2006;14(1):17-30.

67. Völker U, Jacobi C, Trockel MT, Taylor CB. Moderators and mediators of outcome in Internet-based indicated prevention for eating disorders. *Behav Res Ther.* 2014;63:114-121.

68. Zabinski MF, Wilfley DE, Calfas KJ, Winzelberg AJ, Taylor CB. An interactive psychoeducational intervention for women at risk of developing an eating disorder. *J Consult Clin Psychol.* 2004;72(5):914-919.

69. Zabinski MF, Wilfley DE, Pung MA, Winzelberg AJ, Eldredge K, Taylor CB. An Interactive Internet-Based Intervention for Women at Risk of Eating Disorders: A Pilot Study. *Int J Eat Disord.* 2001;30(2):129-137.

70. Mensorio MS, Cebolla-Marti A, Rodilla E, et al. Analysis of the efficacy of an internet-based self-administered intervention ("Living Better") to promote healthy habits in a population with obesity and hypertension: An exploratory randomized controlled trial. *Int J Med Inform.* 2019;124:13-23.

71. Winzelberg AJ, Eppstein D, Eldredge KL, et al. Effectiveness of an Internet-Based Program for Reducing Risk Factors for Eating Disorders. *J Consult Clin Psychol.* 2000;68(2):346-350.

72. Völker U, Jacobi C, Taylor CB. Adaptation and evaluation of an Internet-based prevention program for eating disorders in a sample of women with subclinical eating disorder symptoms: A pilot study. *Eat Weight Disord.* 2011;16(4):270-273.

73. Mason AE, Jhaveri K, Cohn M, Brewer JA. Testing a mobile mindful eating intervention targeting craving-related eating: feasibility and proof of concept. *J Behav Med.* 2018;41(2):160-173.

74. Jacobi C, Morris L, Beckers C, et al. Reduktion von Risikofaktoren für gestörtes Essverhalten: Adaptation und erste Ergebnisse eines Internet-gestützten Präventionsprogramms. *Z Gesundheitspsychol.* 2005;13(2):92-101.

75. Jacobi C, Völker U, Trockel MT, Taylor CB. Effects of an Internet-based intervention for subthreshold eating disorders: a randomized controlled trial. *Behav Res Ther.* 2012;50(2):93-99.

76. Moher D, Schulz KF, Altman DG. The CONSORT statement: revised recommendations for improving the quality of reports of parallel-group randomised trials. *Lancet.* 2001;357(9263):1191-1194.

77. McGoey T, Root Z, Bruner MW, Law B. Evaluation of physical activity interventions in youth via the Reach, Efficacy/Effectiveness, Adoption, Implementation, and Maintenance (RE-AIM) framework: A systematic review of randomised and non-randomised trials. *Prev Med.* 2015;76:58-67.

78. Blackman KCA, Zoellner J, Berrey LM, et al. Assessing the Internal and External Validity of Mobile Health Physical Activity Promotion Interventions: A Systematic Literature Review Using the RE-AIM Framework. *J Med Internet Res.* 2013;15(10):81-95.

79. Wade TD, Wilksch SM. Internet eating disorder prevention. *Curr Opin Psychiatry.* 2018;31(6):456-461.

80. Glasgow RE, Huebschmann AG, Brownson RC. Expanding the CONSORT Figure: Increasing Transparency in Reporting on External Validity. *Am J Prev Med.* 2018;55(3):422-430.

81. Fleming T, Bavin L, Lucassen M, Stasiak K, Hopkins S, Merry S. Beyond the trial: systematic review of real-world uptake and engagement with digital self-help interventions for depression, low mood, or anxiety. *J Med Internet Res.* 2018;20(6):e199.

82. Kohl LFM, Crutzen R, de Vries NK. Online Prevention Aimed at Lifestyle Behaviors: A Systematic Review of Reviews. *J Med Internet Res.* 2013;15(7):71-83.

83. Beintner I, Vollert B, Zarski A-C, et al. Adherence Reporting in Randomized Controlled Trials Examining Manualized Multisession Online Interventions: Systematic Review of Practices and Proposal for Reporting Standards. *J Med Internet Res.* 2019;21(8).

84. Beatty L, Binnion C. A Systematic Review of Predictors of, and Reasons for, Adherence to Online Psychological Interventions. *Int J Behav Med.* 2016;23(6):776-794.

85. Taylor CB, Ruzek JI, Fitzsimmons-Craft EE, et al. Using Digital Technology to Reduce the Prevalence of Mental Health Disorders in Populations: Time for a New Approach. *J Med Internet Res.* 2020;22(7).

86. Hone LC, Jarden A, Schofield GM. An evaluation of positive psychology intervention effectiveness trials using the re-aim framework: A practice-friendly review. *J Posit Psychol.* 2015;10(4):303-322.

87. Baumeister H, Reichler L, Munzinger M, Lin J. The impact of guidance on Internet-based mental health interventions—A systematic review. *Internet Interv.* 2014;1(4):205-215.

88. Kass AE, Balantekin KN, Fitzsimmons-Craft EE, Jacobi C, Wilfley DE, Taylor CB. The economic case for digital interventions for eating disorders among United States college students. *Int J Eat Disord.* 2017.

89. Bennett K, Bennett AJ, Griffiths KM. Security considerations for e-mental health interventions. *J Med Internet Res.* 2010;12(5):e61.

90. Loucas CE, Fairburn CG, Whittington C, Pennant ME, Stockton S, Kendall T. E-therapy in the treatment and prevention of eating disorders: A systematic review and meta-analysis. *Behav Res Ther.* 2014;63:122-131.

91. Mohr DC, Lyon AR, Lattie EG, Reddy M, Schueller SM. Accelerating Digital Mental Health Research From Early Design and Creation to Successful Implementation and Sustainment. *J Med Internet Res.* 2017;19(5):e153.

92. Omimo A, Taranta D, Ghiron L, et al. Applying ExpandNet's systematic approach to scaling up in an integrated population, health and environment project in East Africa. *Social Sciences.* 2018;7(1):8.
